# Pyramidal cell types drive functionally distinct cortical activity patterns during decision-making

**DOI:** 10.1038/s41593-022-01245-9

**Published:** 2023-01-23

**Authors:** Simon Musall, Xiaonan R. Sun, Hemanth Mohan, Xu An, Steven Gluf, Shu-Jing Li, Rhonda Drewes, Emma Cravo, Irene Lenzi, Chaoqun Yin, Björn M. Kampa, Anne K. Churchland

**Affiliations:** 1 Institute of Biological Information Processing (IBI-3), Forschungszentrum Jülich, Jülich, Germany; 2 Department of Systems Neurophysiology, Institute for Zoology, RWTH Aachen University, Aachen, Germany; 3 Cold Spring Harbor Laboratory, Neuroscience, Cold Spring Harbor, New York, NY USA; 4 Department of Neurosurgery, Zucker School of Medicine at Hofstra/Northwell, Hempstead, NY USA; 5 Department of Neurobiology, Duke University Medical Center, Durham, NC USA; 6 Department of Neurobiology, David Geffen School of Medicine, University of California, Los Angeles, Los Angeles, CA USA; 7 JARA Brain, Institute for Neuroscience and Medicine (INM-10), Forschungszentrum Jülich, Jülich, Germany

**Keywords:** Decision, Perception, Cellular neuroscience

## Abstract

Understanding how cortical circuits generate complex behavior requires investigating the cell types that comprise them. Functional differences across pyramidal neuron (PyN) types have been observed within cortical areas, but it is not known whether these local differences extend throughout the cortex, nor whether additional differences emerge when larger-scale dynamics are considered. We used genetic and retrograde labeling to target pyramidal tract, intratelencephalic and corticostriatal projection neurons and measured their cortex-wide activity. Each PyN type drove unique neural dynamics, both at the local and cortex-wide scales. Cortical activity and optogenetic inactivation during an auditory decision task revealed distinct functional roles. All PyNs in parietal cortex were recruited during perception of the auditory stimulus, but, surprisingly, pyramidal tract neurons had the largest causal role. In frontal cortex, all PyNs were required for accurate choices but showed distinct choice tuning. Our results reveal that rich, cell-type-specific cortical dynamics shape perceptual decisions.

## Abstract (web summary)

Distinct cortical pyramidal neuron types, defined by developmental lineage, make unique contributions to behavioral decisions. We highlight the importance of interactions among diverse cortical and subcortical areas for successful decision outcomes.

## Main

The neocortex is organized into discrete layers that form a conserved microcircuit motif. Each layer consists of distinct cell types that can be categorized by genetic markers, morphology, anatomical projections or developmental lineage^1^. The precise interplay across cell types is crucial for cortical computations and their functional roles are intensely studied. For cortical interneurons, cell-type-specific mouse lines have enabled tremendous progress and revealed the functional arrangement of inhibitory circuit motifs^2–4^, for example, for network synchronization^5–7^ and state-dependent sensory processing^8–11^. However, the roles of glutamatergic PyN types are less well established, although PyNs make up ~80% of all cortical neurons and form almost all long-range projections that enable the communication between cortex and other brain areas.

While often treated as a monolithic group, PyNs are far more diverse than interneurons. RNA sequencing indicates at least 100 putative subtypes that are often intermingled within the same layers^12–17^. PyNs are also broadly categorized into two major types based on their long-range projections: intratelencephalic (IT) neurons project to other cortical areas and the striatum, while pyramidal tract (PT) neurons project to subcortical structures, such as the pons and thalamus. PT and IT neurons also differ in their electrophysiological properties, dendritic arborization, local connectivity and sensory tuning^15–17^. Moreover, only PT neurons in sensory cortex are required for perception of tactile or visual stimuli, suggesting that PT and IT neurons encode separate streams of information^18,19^. Correspondingly, specific PT neurons in secondary motor cortex (M2) are involved in motor generation^13,20^. The functional divergence of PyN types could therefore be key for understanding cortical microcircuits, with PT and IT neurons forming functionally distinct, parallel subnetworks that independently process different information. However, the functional tuning of individual PyNs in frontal cortex remains best predicted by cortical area location instead of laminar location or projection type^21^. Because PyN-type-specific activity has only been studied in single areas, it is therefore not known whether PyN-specific subcircuits are the rule or the exception across cortical areas.

An ideal method to address this question is widefield calcium imaging, allowing neural measures across the dorsal cortex with cell-type specificity^22–24^. Interneuron-specific widefield imaging revealed distinct spatiotemporal dynamics for different inhibitory cell types during an odor detection task^25^. However, cortex-wide studies of different PyN types are lacking, partly due to the limited availability of PyN-specific driver lines^26–28^. Here, we used two novel knock-in lines^26^ and measured cortex-wide PT or IT activity while animals performed a perceptual decision-making task. Moreover, we used retrograde labeling to measure the activity of corticostriatal (CStr) projection neurons throughout the dorsal cortex. Dimensionality-reduction and clustering analyses revealed unique cortex-wide dynamics for each PyN type, suggesting the existence of specialized subcircuits. Cortical dynamics of different PyNs were further segregated based on their role in decision-making, with encoder and decoder approaches revealing the strongest stimulus-related and choice-related modulation in sensory, parietal and frontal cortices. This was confirmed by PyN-type-specific inactivation experiments. In parietal cortex, PT neurons were most important for sensory processing, while all PyN types in frontal cortex were needed for choice formation and retention. Taken together, our results demonstrate that different PyN types exhibit functionally distinct, cortex-wide neural dynamics with separate roles during perceptual decision-making.

## Results

### Pyramidal tract and intratelencephalic neurons show distinct cortex-wide activity patterns

**Fig. 1:**
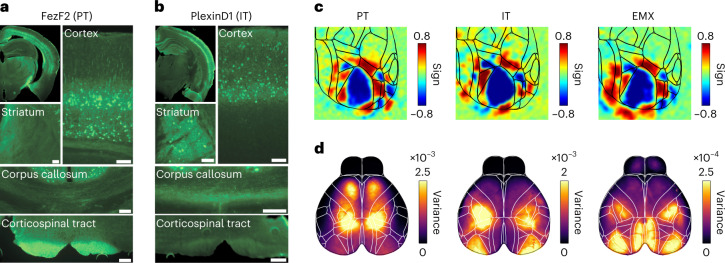
Knock-in mouse lines enable pyramidal neuron subtype-specific recordings of cortex-wide neural activity. **a**, GCaMP6s expression in Fezf2-2A-CreER;Ai162 mice. Cortical labeling is largely confined to layer 5b. Axonal projections were found in multiple subcortical regions such as the striatum and the corticospinal tract. Scale bars, 100 µm. **b**, GCaMP6s expression in PlexinD1-2A-CreER;Ai162 mice is widespread throughout the cortex and restricted to superficial layers 2/3 and layer 5a. Axonal projections were found in striatum and corpus callosum but absent in the corticospinal tract. **c**, Visual sign maps from retinotopic mapping experiments. IT and PT populations showed clear retinotopic responses in primary and secondary visual areas where boundaries largely resembled known areas from nonspecific PyNs (EMX). **d**, Total variance maps from same mice as in **c**, showing most modulated cortical regions in each PyN type.

We used CreER lines to measure the activity of two developmentally distinct PyN types: Fezf2-CreER targeting PT neurons and PlexinD1-CreER targeting IT neurons^26^, crossed with Ai162 mice^29^ to achieve PyN-type-specific expression of the calcium indicator GCaMP6s. As expected for corticofugal PT neurons, GCaMP expression in Fezf2 mice was concentrated in layer 5b with axonal projections to subcortical regions and the corticospinal tract (Fig. 1a). In PlexinD1 mice, expression was restricted to layers 2/3 and 5a with axonal projections in the corpus callosum and the striatum, matching intracortical and corticostriatal IT neurons (Fig. 1b).

**Fig. 2:**
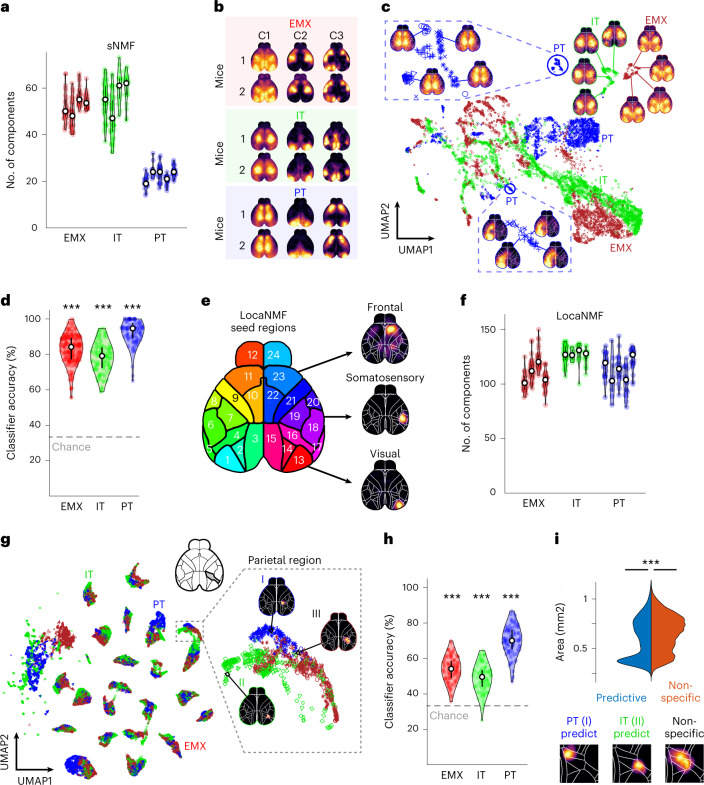
Pyramidal neuron types exhibit unique cortical activity patterns. **a**, Number of sNMF components, accounting for 99% of variance per PyN type. Violin plots show individual mice; dots indicate individual sessions. **b**, Spatial sNMF components from different mice of the same PyN type (colored rectangles) strongly resembled each other. **c**, UMAP embedding of spatial sNMF components for EMX (red), IT (green) and PT (blue) mice. Maps show spatial components at different UMAP locations. Marker types denote individual mice. Blown-up areas show examples of PT-specific regions. **d**, Accuracy of a PyN-type classifier. Data points represent the mean classification accuracy per session. Asterisks represent Bonferroni-corrected *P* < 10^−10^ against 33% chance (two-sided *t*-test, *n*_EMX_ = 124 sessions; n_IT_ = 133, n_PT_ = 93 sessions). **e**, Map of seed regions used for LocaNMF analysis. **f**, Number of LocaNMF components, accounting for 99% of variance per PyN type. Conventions as in **a**. **g**, UMAP projection embedding of spatial LocaNMF components. Conventions as in **c**. The UMAP shows clustering of LocaNMF components from similar regions (same 24 regions as in **e**). Components within individual regions are further divided for different PyN types. Maps show example LocaNMF components (I–III). **h**, Accuracy of a PyN type classifier, based on individual LocaNMF components. Conventions as in **d**. Asterisks denote Bonferroni-corrected *P* < 10^−10^ against 33% chance (two-sided *t*-test, *n*_EMX_ = 124 sessions; *n*_IT_ = 133, *n*_PT_ = 93 sessions). **i**, Peak normalized distributions of area size for PyN-type-predictive (blue) versus nonspecific (red) LocaNMF components. PyN-predictive components are smaller than nonspecific components (PyN-predictive: median = 0.59 mm^2^, *n* = 6,317 components; nonspecific: median = 0.68mm^2^, *n* = 18,938 components; two-sided rank-sum test: *P* < 10^−10^). Examples of PyN-predictive (I and II) and nonspecific (III) components in right parietal cortex.

**Extended Data Fig. 1:**
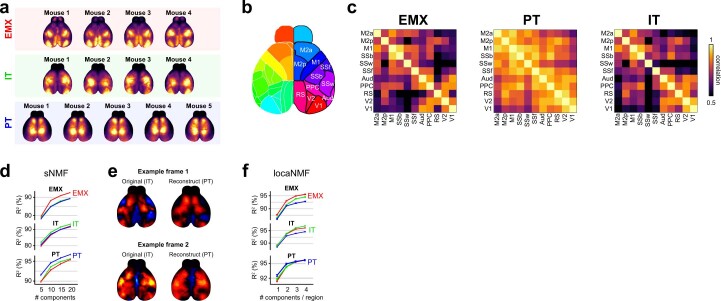
Pyn-specific cortical maps of total variance and inter-regional correlations. (**a**) Maps of variance over all frames for individual mice in each PyN type group. Colors are normalized between zero and the 95th percentile for each animal. Distinct variance patterns for each PyN type were largely conserved across individual mice. (**b**) Map of cortical regions, used for correlation analysis. V1 = primary visual cortex, V2 = secondary visual cortex, RS = retrosplenial cortex, Aud = auditory cortex, PPC = posterior parietal cortex, SSw = somatosensory whisker area, SSb = somatosensory body area, SSf = somatosensory face area, M1 = primary motor cortex, M2p = posterior secondary motor cortex, M2a = anterior secondary motor cortex. (**c**) Correlations between cortical regions in EMX, PT and IT neurons averaged over all sessions and mice. Inter-region correlations were comparable between EMX and IT neurons but overall increased for PT neurons. (**d**) R^2^ of EMX, IT and PT reconstructions (top to bottom panels), using components from different PyN types (red, green, and blue traces). For within-group reconstructions, only components from other mice were used. (**e**) Single-frame reconstructions of IT data, using PT components. IT imaging data (original) was projected onto PT components to assess if they would be applicable to capture IT variance. While the reconstruction captured a large fraction of variance (~93%, Supplementary Video 4, comparing individual frames showed that PT components did not recreate more fine-grained spatial features of the IT data. (**f**) R^2^ of EMX, IT and PT reconstructions, using locaNMF components from different PyN types (formatting as in panel a)). Shown are results for different number of components per region, using 24 regions in total. The minimum number of components was therefore 24 (1 component per region) and the maximum 96 (4 components per region).

We then measured PyN-type-specific cortical activity with widefield imaging. In both lines, we observed rich neural dynamics across cortex (Supplementary Videos 1–3) and retinotopic mapping revealed known visual area locations (Fig. 1c)^30,31^. Retinotopic maps were similar to those in Ai93D;Emx-Cre;LSL-tTA (EMX) mice with nonspecific GCaMP6f expression across PyNs^32^, suggesting that the functional architecture of visual areas is comparable across PyN types. However, the variance of cortical activity was clearly PyN-type specific, being largest in parietal and frontal regions in PT neurons, and visual and somatosensory regions in IT neurons (Fig. 1d). Variance maps were also highly consistent across individual mice in the same group (Extended Data Fig. 1a), indicating PyN-type-specific differences in cortex-wide activity patterns. To isolate activity patterns, we therefore performed semi-nonnegative matrix factorization (sNMF), reducing the imaging data to a small number of spatial and temporal components that capture >99% of all variance^23,33^. Surprisingly, PT neurons had a lower dimension than IT neurons (Fig. 2a), potentially because IT neurons encompass a larger number of specialized subtypes than PT neurons and thus support a wider range of functions^34^.

Next, we tested if spatial sNMF components, representing cortex-wide maps of positively correlated areas, were PyN-type specific. Indeed, components from different mice of the same PyN type strongly resembled each other but differed from other PyN types (Fig. 2b). To assess if most components were PyN-type specific, we performed a uniform manifold approximation and projection (UMAP) analysis of the first 20 components from all recordings, nonlinearly embedding the pixels of each component in a two-dimensional space (Fig. 2c)^35^. In agreement with the notion that components of the same PyN type resembled each other, PT and IT components formed strong clusters (green/blue markers). EMX neurons formed a third set of nonoverlapping clusters, likely reflecting the combined cortical dynamics from diverse PyN types beyond PT and IT neurons (red markers).

A simple classifier reliably identified each group, based on the nearest neighbors in a UMAP projection using data from other animals. Remarkably, even single-component classification achieved very high prediction accuracy, although components were pooled over many sessions and mice (Fig. 2d). UMAP clusters therefore reflect consistent PyN-type-specific activity patterns, rather than idiosyncratic differences or noise. These results clearly demonstrate that PyN types differ in the complexity of cortical dynamics, contain independent variance and exhibit unique cortex-wide correlation patterns.

**Extended Data Fig. 2:**
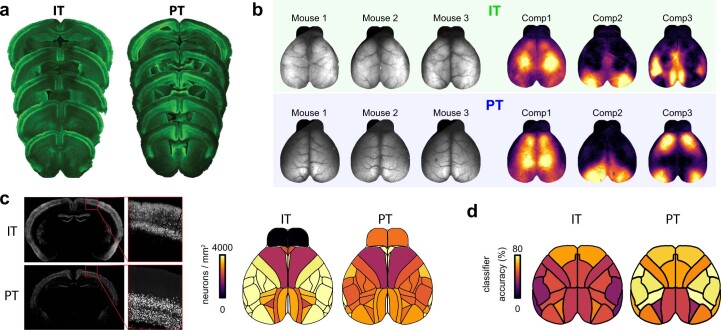
Cortex-wide expression patterns of PT and IT neurons. (**a**) Brain slices from IT and PT neurons show robust cortex-wide expression of GCaMP6. (**b**) Left: Raw fluorescence from widefield imaging of 3 different PT and IT mice. In both lines, we obtained strong fluorescence throughout the cortex, although minor fluctuations in brightness were visible across regions. Right: Example sNMF components from an individual PT/IT mouse (mouse #3 for both lines). sNMF components did not strongly reflect differences in raw fluorescence across cortex. We also observed no clear relationship between fluorescence patterns to total variance (Fig. 1D) or ongoing activity patterns (Supplementary. Movies 2, 3). (**c**) Left: Example brain slices from IT- and PT-Cre mice to quantify the density of Cre-expressing neurons in each line. Blow-up shows a magnified region in cortex with individual somata. Right: Expression density was largely even across dorsal cortex with higher density of IT neurons in lateral regions and no expression in the olfactory bulb. Density was slightly reduced in M2 for both lines. (**d**) Map of PyN type decoding accuracy with locaNMF components for different cortical regions. Decoding accuracy was high across cortical regions and we found no clear relation between expression patterns and regions with particularly high locaNMF decoding accuracy. Olfactory bulb was omitted from the analysis, due to the lack of strong fluorescence signals.

An important concern is that nonuniform Cre expression could contribute to PyN-specific spatial components. However, in vivo GCaMP-related fluorescence was largely uniform, with no clear relationship between widefield fluorescence or PyN-type-specific spatial components and Cre expression patterns (Extended Data Fig. 2). Nevertheless, particularly distinct PyN-type activity in specific cortical areas could lead to cortex-wide correlation patterns either that are dominated by highly active areas or where inactive areas are ‘missing’. We therefore used localized sNMF (LocaNMF)^33^, which extracts components that are restricted to a specific cortical ‘seed’ region (Fig. 2e). Analyzing LocaNMF components therefore allowed us to reveal if PyN-specific differences mostly occur on a cortex-wide level (reflecting the interactions between cortical areas) or extend to specific properties of local cortical areas (reflecting PyN-type-specific differences in the shape or localization of individual areas).

The number of LocaNMF components was greater than sNMF and, interestingly, more similar across PyN types (Fig. 2f). PyN-type-specific differences in cortex-wide correlation patterns are therefore not due to low activity in specific cortical areas (which would reduce the total number of required components, for example, in PT mice) but reflect differences in the coordinated activation of multi-area cortical networks. UMAP embedding of LocaNMF components also uncovered PyN-type-specific clustering (Fig. 2g), which accurately identified each PyN type across most cortical regions (Fig. 2h and Extended Data Fig. 2d). PyN-type specificity of LocaNMF components could indicate either specific ‘subregions’, where PyN types are most active in smaller parts of a given cortical area, or larger ‘superregions’, where PyN-type-specific activity extends across area borders. We compared the size of LocaNMF components that accurately predicted their respective PyN type (classifier accuracy > 99%) versus nonspecific components. Interestingly, PyN-predictive components were significantly smaller than nonspecific components (Fig. 2i), suggesting that different PyNs might be most active in distinct subregions instead of larger multi-area components. This indicates that smaller, PyN-type-specific subregions may reside within the coarser, traditionally defined cortical areas.

### Pyramidal tract and intratelencephalic neurons show distinct task-related activity

**Fig. 3:**
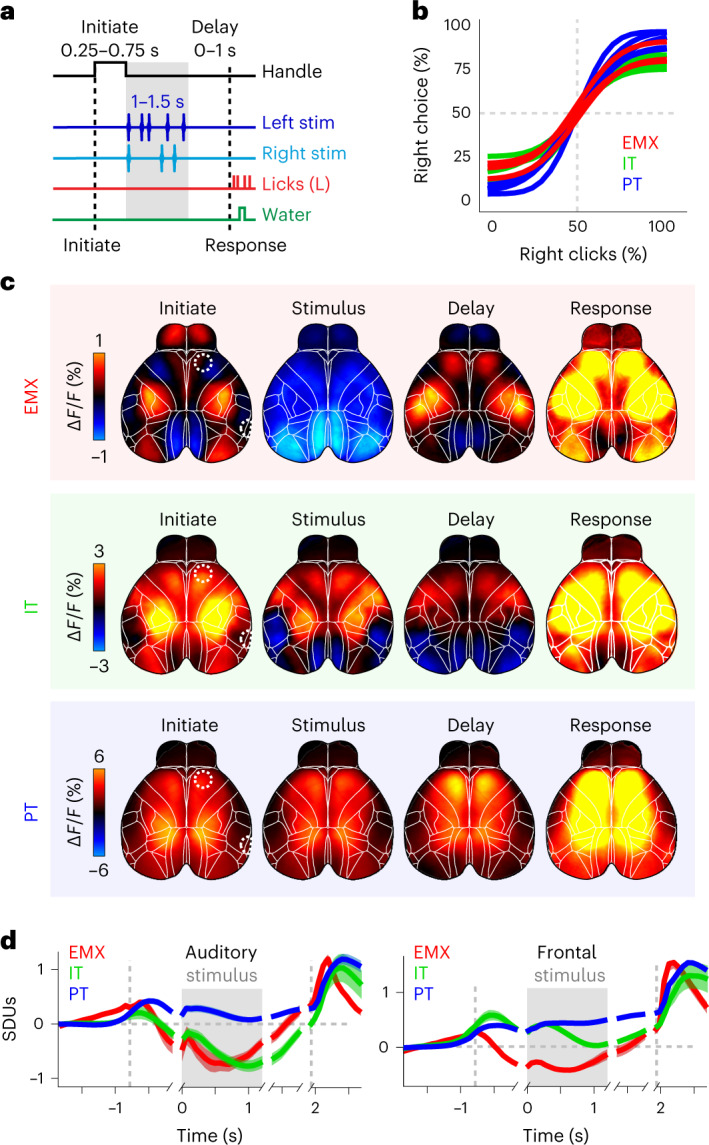
An auditory decision-making task reveals distinct functional activity patterns in each pyramidal neuron type. **a**, Auditory discrimination task structure. Mice touched paw handles to initiate randomized click sequences on the left and/or right side. After a delay period, a lick response on the correct side was rewarded with water. **b**, Psychometric functions fit to behavioral data from the discrimination task in **a** for individual EMX (red), IT (green) and PT (blue) mice. **c**, Trial-averaged response maps for all correct, leftward trials in different PyN types. Shown are averages for the ‘initiation’, ‘stimulus’, ‘delay’ and ‘response’ periods in **a**. **d**, Averaged activity of each PyN type in auditory (left) and frontal cortex (right) (dashed circles in **c**). Averages were separately aligned to each of the four trial periods, indicated by short gaps. The left dashed line indicates time of initiation, the gray box indicates stimulus presentation and the right dashed line indicates the animal’s response. Traces show standard deviation units (SDUs). Colors as in **b**. Shading shows the s.e.m.; *n*_EMX_ = 4, *n*_IT_ = 4, *n*_PT_ = 5 mice. Δ*F/F*, fluorescence intensity change.

**Extended Data Fig. 3:**
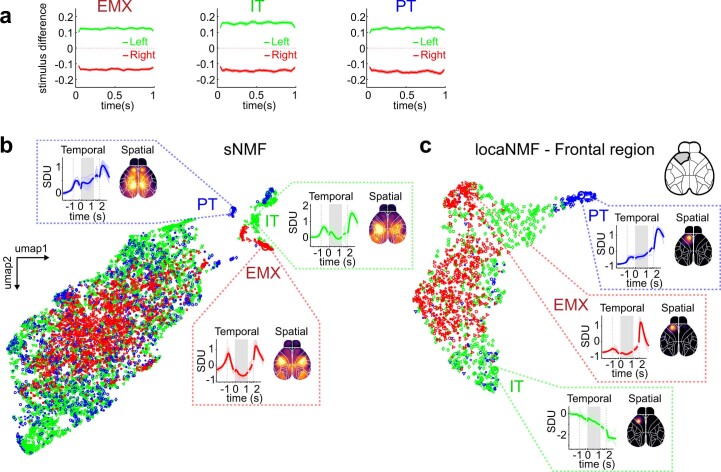
Temporal structure in animal behavior and cortical activity patterns. (**a**) Mice in all PyN groups integrate sensory information throughout the stimulus period. Shown is the normalized difference between auditory clicks on the left or right side, when animals successfully responded to the left (green) or the right (red). Binsize is 50 ms. Positive numbers indicate a higher probability of observing a leftward click sound, negative numbers indicate more clicks on the right. In all mice, the probability of observing more stimuli on the correct side is consistently higher throughout the stimulus period. This shows that mice integrate sensory evidence from the entire stimulus period and auditory clicks equally influence animal decisions, regardless of whether they occur early or late in the stimulus sequence. (**b**) Clustering of temporal components. UMAP embedding of temporal sNMF components for EMX (red), IT (green) and PT (blue) mice. Clustering for cell types is weaker as with spatial components (Fig. 2c) but clearly visible, suggesting that sNMF components are both spatially and functionally distinct. Insets show 10 example traces of trial-averaged activity from cell-type specific clusters (left, bold line shows the mean) and an example of a corresponding spatial component (right). (**c**) UMAP embedding of temporal locaNMF components from left frontal cortex. Conventions as in a). Temporal locaNMF components also show cell- type- specific clustering, revealing task-specific dynamics (inset, left). Spatial locaNMF components also show separate shapes for each cell type (inset, right).

We next assessed functional PyN-type-specific cortical dynamics by imaging animals during auditory decision-making (Fig. 3a)^36^. Mice touched small handles, triggering the simultaneous presentation of click sequences to their left side and right side. After a delay, choosing one of two water spouts was rewarded when licking on the side where more clicks were presented. To reduce temporal correlations across task events, the durations of the initiation, stimulus and delay periods were randomly varied across trials. In all mice, decisions varied systematically with stimulus strength (Fig. 3b) and were equally affected by clicks throughout the stimulus period (Extended Data Fig. 3a).

**Extended Data Fig. 4:**
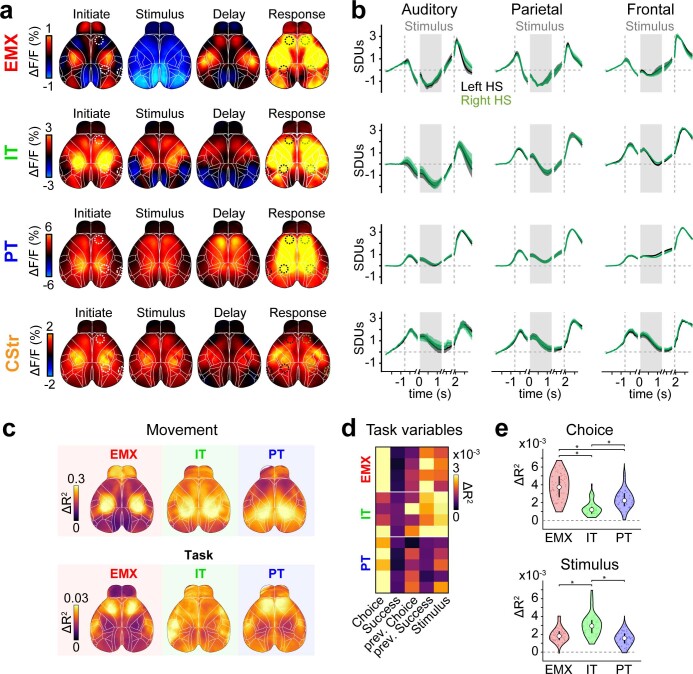
Symmetric bilateral activation during decision-making and unique explained variance. (**a**) Trial-averaged response maps for all correct, leftward trials across different PyN types. Cortical maps are as shown in Fig. 3a and Fig. 7c. (**b**) Average activity in auditory, parietal, and frontal cortex on the left (black) and right hemisphere (green), which are contra- and ipsilateral to the chosen side, respectively. In all PyN types, different trial events, such as initiation, sensory stimulation and animal responses increased neural activity. However, surprisingly few differences were seen between cortical hemispheres. To resolve differences in inter-hemispheric activation for left- versus rightward choices we therefore employed the choice decoder analysis (Figs. 6 and 7). Note that low or negative weights from the choice decoder (as seen for IT and CStr neurons in frontal cortex) do not reflect a lack of choice-related activity activity but are rather based on small differences in the activation of hemispheres that are either ipsi- or contralateral to the chosen side. (**c**) To isolate unique contributions from movement or task variables, we computed averaged maps of the loss in predicted variance (ΔR^2^) by removing either group of variables from the full model. This allowed us to separately examine their respective impact on cortex-wide activity by determining, for each PyN type, where in the cortex predictive power was lost. While movement ΔR^2^ patterns were comparable across PyN types (top row), PyN-type-specific differences were uncovered when removing task variables: ΔR^2^ was highest in frontal cortex of EMX and PT mice, but more diffuse in IT mice with the highest ΔR^2^ in auditory cortex (bottom row). Note differences in scale between two rows. (**d**) Examination of ΔR^2^ for individual task variables further suggest distinct roles for each PyN type. Here, the ‘choice’ variable had the highest contributions in PT neurons but was overall weaker in IT neurons. Conversely, contributions from other task variables were higher in IT neurons. This dichotomy was not observed in EMX neurons, indicating that IT and PT neurons might have different functional roles that cannot be resolved without PyN-type specific measurements. Each row represent a mouse. (**e**) Comparison of ΔR^2^ for choice (top) and stimulus variables (bottom) between PyN types. IT mice had significantly lower ΔR^2^ for choice (*p*_*EMX*_ = 1.4 × 10^−22^, *p*_*IT*_ = 1.1 × 10^−7^; n_EMX_ = 62, n_IT_ = 71, n_PT_ = 59 sessions) but higher ΔR 2 for the stimulus as EMX or PT mice (*p*_*EMX*_ = 1.2 × 10^−5^, *p*_*IT*_ = 4.9 × 10^−14^). Note that lower ΔR^2^ for choice in IT mice does not imply a lack of involvement in decision formation but rather that their population activity does not clearly differ for left versus right choices. Dots indicate individual sessions. Stars indicate significant differences across sessions (two-sided unpaired *t*-test, p < 0.01, bonferroni-corrected).

Trial-averaged temporal sNMF and LocaNMF components also showed pronounced clustering, suggesting that PyN types exhibit unique task-related temporal dynamics (Extended Data Fig. 3b). Correspondingly, trial-averaged neural activity between PyN types was clearly distinct, especially during stimulus presentation when EMX activity was uniformly suppressed, IT activity was partially suppressed in somatosensory and visual cortex, and PT activity was uniformly elevated (Fig. 3c,d). Cortical activity was largely symmetric between hemispheres, even when only analyzing trials where both stimuli and subsequent choices were leftward (Extended Data Fig. 4). Moreover, stimulus responses were much weaker than movement-related activity, such as trial initiation or licking (Fig. 3d). Lateralized, task-related activity could have therefore been obscured by cortical activity due to animal movements^32,37–39^.

**Fig. 4:**
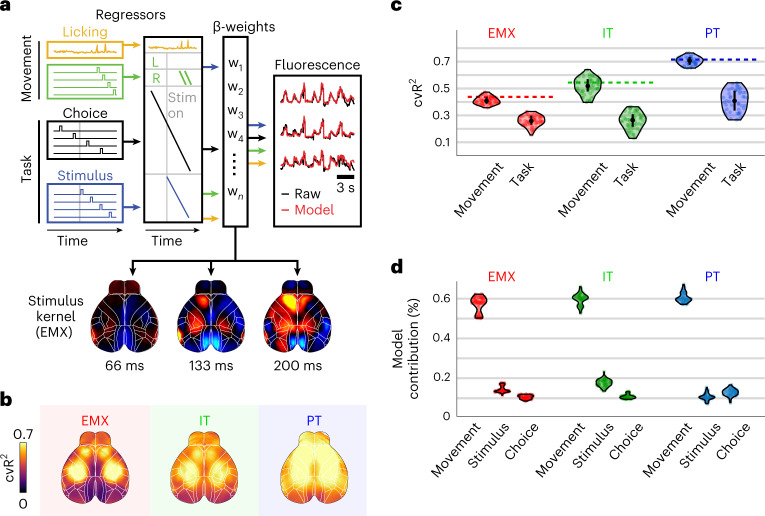
An encoding model uncovers task-specific differences across pyramidal neuron types. **a**, Schematic of the encoding model. Top, task-related and movement-related variables account for fluctuations in cortical activity. Bottom, weights for each variable define a spatiotemporal event kernel, revealing cortical activity in response to a specific event (example shows right stimulus kernel in EMX mice). **b**, Average maps of cvR^2^. The model accurately predicted cortical variance for all PyNs, with over 90% explained variance in the frontal cortex of PT mice. **c**, cvR^2^ from two models, using only movement (‘Movement’) or task (‘Task’) variables. In all groups, movements were more predictive than task variables and accounted for the majority of the explained variance of the full model (dashed lines). Circles denote sessions. **d**, Contributions of movements, stimulus and choice to the model’s total explained variance. Although movements contributed the most, stimulus and choice also made sizable contributions.

To isolate task-related activity, we used a linear encoding model, combining many task-related and movement-related variables (Supplementary Table 1) to predict single-trial fluctuations in cortical activity (Fig. 4a)^32^. Task variables included sensory stimuli, and past and current choices. Movement variables included licking, handle touch or facial movements (see Methods for a complete variable list). After combining all variables and fitting the model, we obtained time-varying event kernels, showing how each variable (for example, the sensory stimulus) relates to cortical activity. This allowed us to separate task-related and movement-related activity.

To assess the accuracy of model predictions, we computed the cross-validated explained variance (cvR^2^). Across PyNs, the model captured a large fraction of single-trial variance throughout dorsal cortex (Fig. 4b) and, consistent with earlier results^32,37^, movements captured more variance than task variables (Fig. 4c). We then focused on the event kernels for stimulus and choice, to reveal their respective PyN-type-specific cortical dynamics. To ensure that stimulus and choice accounted for a sizable amount of the neural activity, we computed the variance that each kernel contributed to the full model compared to the sum of all movement variables (Fig. 4d). While movement variables made the largest model contributions (~60% explained variance), both stimulus and choice also made sizable contributions (10–20% explained variance). Stimulus and choice therefore remain important for understanding cortical activity patterns and can be leveraged to selectively isolate task-related activity.

**Fig. 5:**
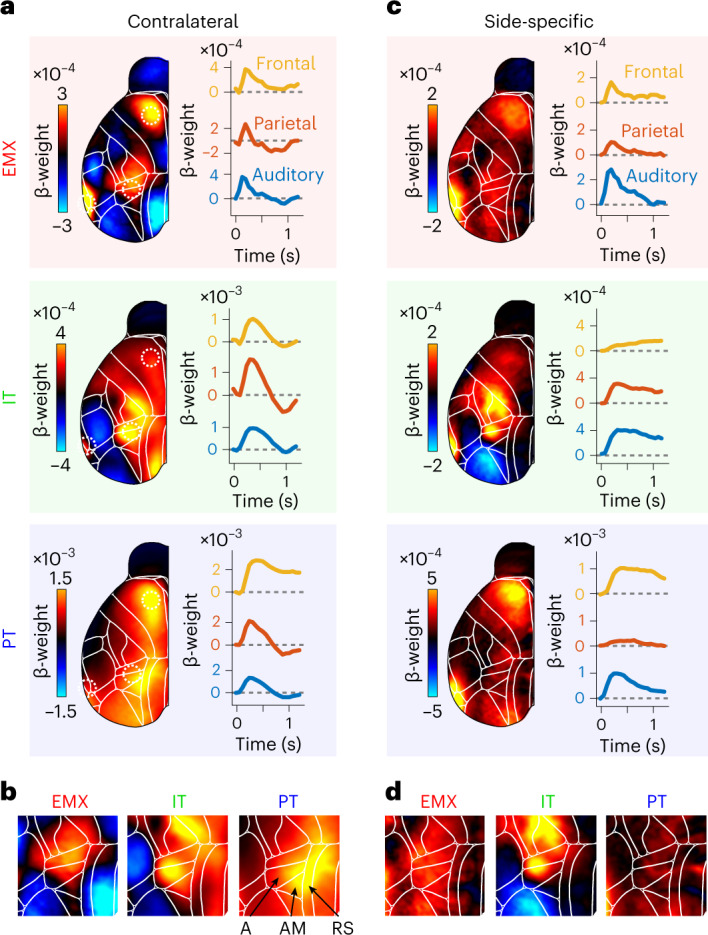
Pyramidal neuron-specific differences are evident in the location and specificity of cortical stimulus responses. **a**, Left, response kernels for contralateral stimuli in EMX (red), IT (green) and PT neurons (blue), averaged between 0 and 200 ms across all mice per group. Right, stimulus-evoked activity in auditory (blue), parietal (red) and frontal cortices (yellow). Dashed circles on the left show cortical locations. **b**, Magnified view of parietal cortex for stimulus maps in **a**. PyNs differed in the location of sensory responses. Arrows show location of parietal areas A, AM and retrosplenial cortex (RS; Allen Brain Atlas, CCF v3)^31^. **c**, Side-specific stimulus responses, computed as the difference between contralateral and ipsilateral stimulus kernels. Hot colors denote stronger contra-response. Conventions as in **a**. **d**, Magnified view of parietal cortex for side-specific maps in **c**. IT neurons show clear, side-specific parietal responses that were weaker in EMX and absent for PT neurons.

We first investigated responses to the auditory stimulus. In contrast to trial averages of Δ*F/F* (Fig. 3c), EMX stimulus kernels uncovered lateralized responses in auditory, parietal and frontal cortex while somatosensory and visual cortex, were inhibited (Figs. 4a and 5a). Sensory-locked responses were also present in auditory, parietal and frontal cortices of PT and IT mice but no inhibition was apparent in PT mice. Sensory responses were particularly PyN-type-specific in the parietal cortex: EMX and IT responses were localized in area A, while PT responses were most prominent at the border between areas AM and RS (Fig. 5b). While some areas, such as auditory cortex, preferentially responded to contralateral stimuli, PT neurons in parietal cortex were activated bilaterally in response to ipsilateral or contralateral stimuli. To assess such side-specificity, we subtracted ipsilateral from contralateral stimulus kernels (Fig. 5c). EMX responses were lateralized in auditory, frontal, and to a lesser extent parietal cortex (Fig. 5c,d). Lateralized IT responses were found in auditory and parietal but not frontal cortex. In contrast, PT responses were lateralized in auditory and frontal but not in parietal cortex. Such differences in unilateral versus bilateral responses in PT and IT neurons may also reflect divergent functional roles, with unilateral responses encoding the spatial location of sensory information and bilateral responses representing stimulus salience.

### Pyramidal neuron-type-specific choice signals in frontal cortex

**Fig. 6:**
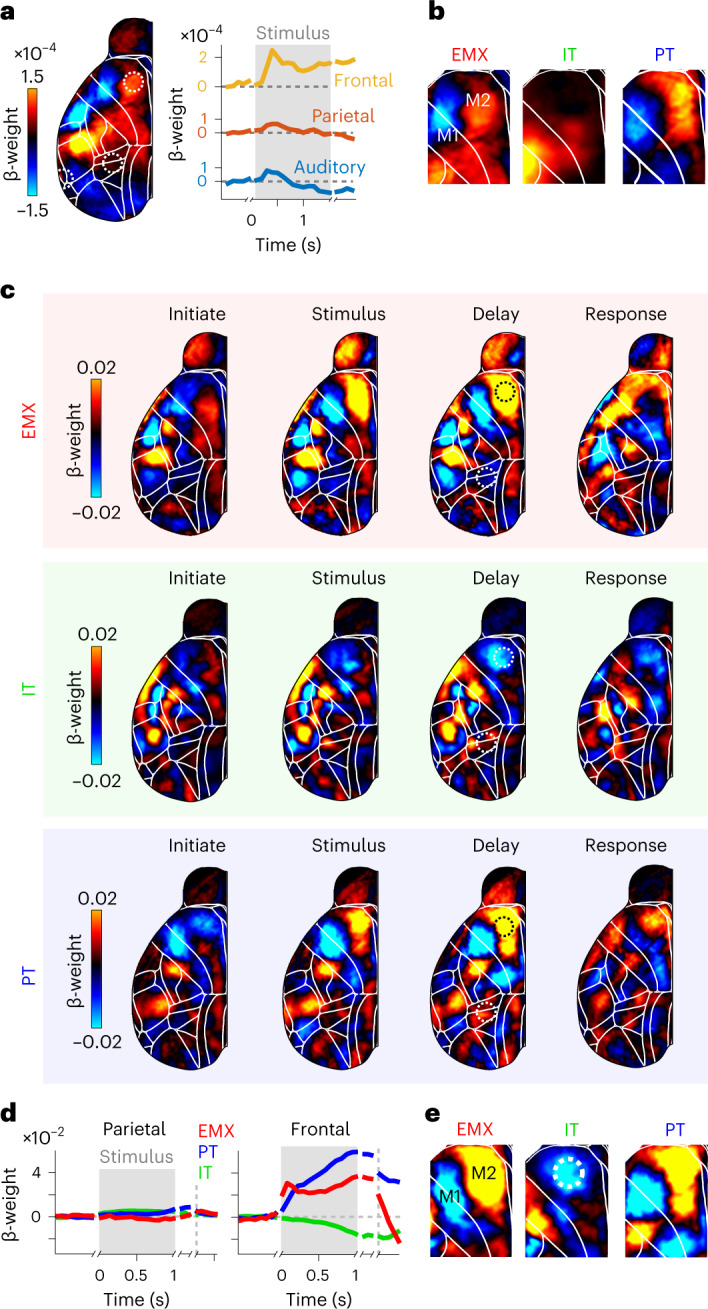
The temporal dynamics of choice-related activity differ across pyramidal neuron types. **a**, Left, averaged contralateral choice kernels for EMX mice during the delay period. Positive weights indicate increased choice-related activity for contralateral choices, while negative weights indicate decreased choice-related activity. Right, choice-related activity in auditory (blue), parietal (red) and frontal cortices (yellow). Traces are realigned to the initiation, stimulus, delay and response periods, indicated by gaps in weight traces. **b**, Zoomed-in map for delay-period frontal choice kernels of EMX, IT and PT neurons. **c**, Cortical maps of contralateral choice weights for different trial episodes. Several areas in anterior cortex showed clear choice signals. **d**, Baseline-corrected decoder weights in parietal (left) and frontal (right) cortices throughout the trial. Conventions as in **a**. Dashed circles in the delay maps of **c** show the parietal and frontal locations used to compute the traces. **e**, Zoomed-in map for frontal delay-period decoder weights of EMX, IT and PT mice. Dashed circle shows the ALM.

**Extended Data Fig. 5:**
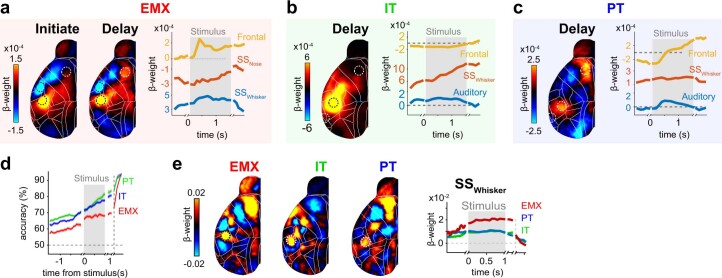
Choice-related activity in somatosensory cortex. (**a**) Averaged choice kernel maps for EMX mice during the initiation and delay period. Dashed circles show location of somatosensory whisker (SS_Whisker_), somatosensory nose (SS_Nose_), and frontal cortex. SS_Whisker_ (blue trace) and SS_Nose_ (red trace) were constantly positive or negative, respectively, even during the initiation period. In contrast to frontal cortex (yellow trace), both areas were only weakly modulation by the stimulus onset (gray box). (**b**) Choice kernel maps for IT mice during the delay period. Dashed circles show location of auditory, SS_Whisker_, and frontal cortex. Choice-related activity in SS_Whisker_ (red trace) increased over the course of the trial. No choice-related modulation was apparent in frontal cortex. (**c**) Choice kernel maps for PT mice during the delay period. Conventions as in (**b**). Choice-related activity strongly increased in frontal cortex after stimulus onset and was weaker in other cortical areas. (**d**) 10x cross-validated decoder performance, predicting animal’s left/right choices at different times during the trial. In all PyN types, decoder performance was above chance at all times, including the initiation period before the stimulus (gray box). This suggests that, in some trials, animals follow a pre-conceived choice that is stimulus-independent and can be decoded from cortical activity. Decoder performance was highest in the response period (dashed vertical line) when animals performed licking movements. (**e**) Contralateral choice weight maps during the delay period (same as in Fig. 6c). Dashed circles show the location of somatosensory whisker cortex (SS_Whisker_). In all PyN types, choice weights in SS_Whisker_ were increased in the initiation period before the stimulus (gray box). A potential explanation could be that pre-stimulus choices are reflected in choice-specific whisker movements. However, choice signals in SS_Whisker_ persisted when removing movement-related activity from the imaging data (Supplementary Fig. 12). Whisker or other facial movements might therefore be too subtle to be captured by our analysis or choice signals in SS_Whisker_ reflect non-overt choice-related activity.

Having identified PyN-type-specific sensory responses, we then examined choice-dependent activity and again observed clear differences across PyN types. In EMX mice, choice-related activity was strongest in the frontal cortex, while sensory and parietal regions were only weakly modulated (Fig. 6a). We also found choice signals in whiskers and nose somatosensory areas that slowly increased during the trial (Extended Data Fig. 5a), potentially because of subtle, choice-predictive whisker or facial movements^40^. In contrast, frontal choice-specific activity strongly increased after stimulus onset and remained elevated into the delay period (Fig. 6a). While PT neurons showed similarly robust choice signals, there was little evidence of IT choice activity (Fig. 6b and Extended Data Fig. 5b,c). In EMX and PT mice, positive contralateral choice signals were concentrated in the medial M2 with some inhibition in primary motor cortex (M1). This could indicate accumulation of sensory evidence and motor preparation in M2, and inhibition in parts of M1 when early lick responses must be witheld^41^.

**Extended Data Fig. 6:**
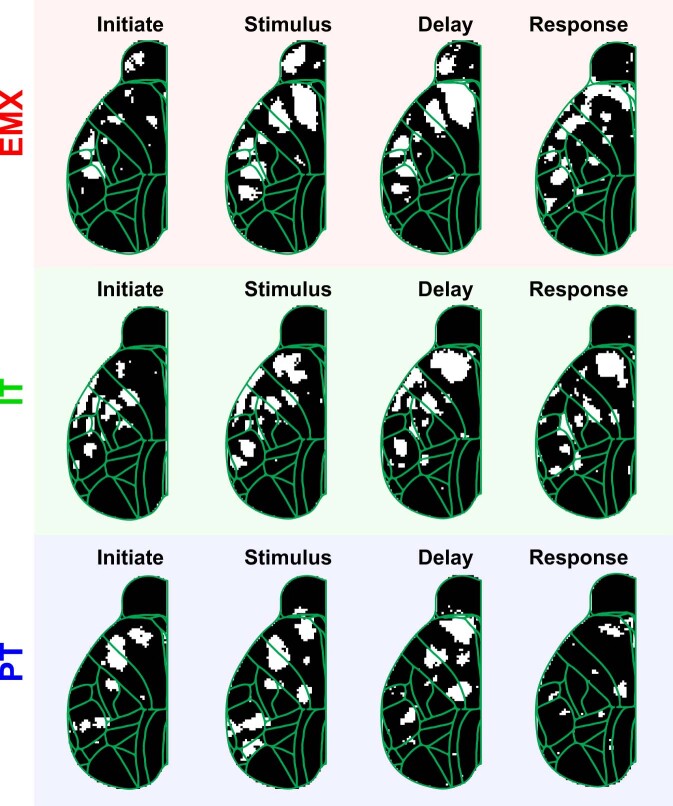
Significance of choice decoder weights. To assess significant weights of the choice decoder, we combined spatially downsampled choice maps from all sessions in each PyN type and subsequently performed a *t*-test in each pixel to determine which decoder weights are significantly different from zero. The resulting maps show significant pixels for different trial periods in white (*t*-test, p < 0.05, bonferroni-corrected for 3364 pixels). Significant pixels closely match choice decoder weights (Fig. 6c) with significant regions being largely tied to anterior cortex. In all PyN types, weights in frontal cortex are significant in the stimulus and delay period, thus supporting the main conclusions of the choice kernel analysis.

**Extended Data Fig. 7:**
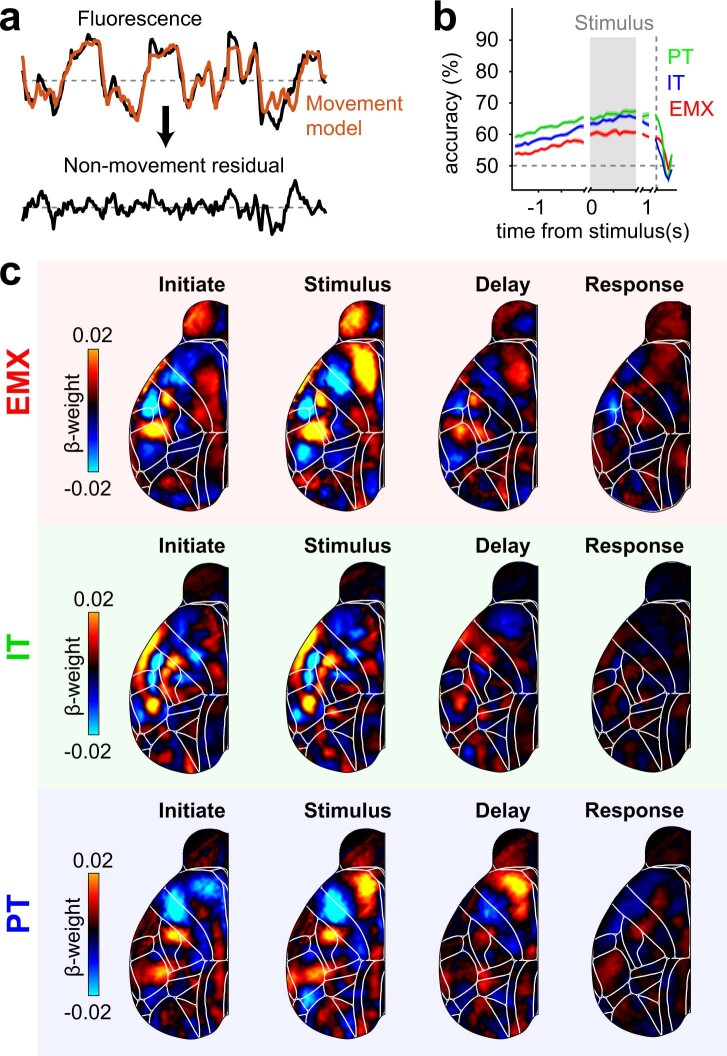
Movement-corrected choice decoder. (**a**) Using a model based on movement variables, we subtracted all movement-related activity from raw fluorescence data and applied the choice decoder analysis to the resulting residuals. (**b**) Removing movement-related activity reduced choice prediction accuracy, in particular during the delay and response period when most choice-related movements occur. In all PyN types, predictions remained above chance levels, suggesting that part of the choice-related activity is independent of observable movements. (**c**) Movement-corrected choice kernels revealed the same cortical patterns as seen in the regular choice decoder (Fig. 6c), demonstrating that choice signals are not solely driven by movements.

Although choice kernels revealed PyN-type-specific differences, they only accounted for a small amount of the total neural variance (Fig. 4d). Because the encoding model maximizes explained variance, we hypothesized that it might miss specific but low-magnitude choice signals. To isolate all choice-related activity, we therefore used a logistic regression classifier with L1 penalty. In contrast to the encoding model, this decoder approach isolates cortical signals that are best suited to predict choices, regardless of their magnitude. Across PyNs, the decoder predicted trial-by-trial choices with high accuracy (Extended Data Fig. 5d). When analyzing the decoder weights, we found comparable patterns to the encoding model’s choice kernels but with much clearer separation of cortical areas (compare the top row for ‘Delay’ in Fig. 6c to left of Fig. 6a). Here, positive decoder weights denote areas that are most predictive for contralateral choices but, importantly, this does not suggest that these areas are necessarily the most active. We found substantial choice signals in multiple areas of the anterior cortex that evolved during decisions (Fig. 6c and Extended Data Figs. 5e and 6). In EMX and PT mice (top and bottom rows), large parts of M2 were again highly choice predictive, including the anterior lateral motor cortex (ALM) and the medial motor cortex (MM)^21^. M2 choice weights strongly increased immediately after stimulus onset and remained elevated during the subsequent delay period (Fig. 6c,d). Additionally, cortical choice signals persisted after removing movement-related activity from the data, suggesting that they are not explained by choice-predictive animal movements but instead reflect the formation of sensory-driven decisions in frontal cortex (Extended Data Fig. 7).

Surprisingly, we also found a mild ipsilateral choice preference for M2 in IT mice, despite strong bilateral activation of frontal cortex during the delay period (Fig. 3c). Ipsilateral choice signals evolved more slowly during the stimulus and delay periods (Fig. 6c,d) and were spatially restricted to the ALM (Fig. 6e). No choice signals were seen in parietal cortex of any PyN type (Fig. 6d), suggesting that parietal cortex is mostly involved in sensory processing instead of choice formation or motor execution^42,43^.

### Corticostriatal projections neurons are a functionally divergent intratelencephalic subclass

**Fig. 7:**
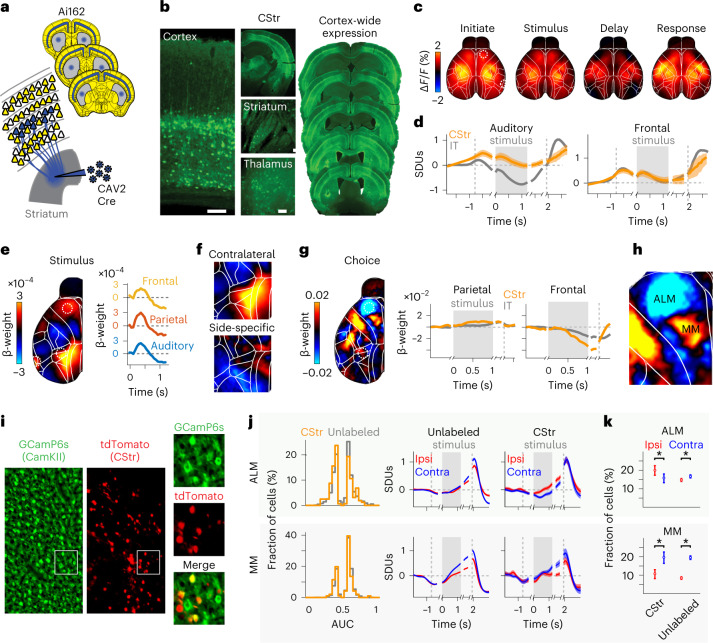
An intersectional approach to measure cortex-wide activity of corticostriatal neurons. **a**, Retrograde CAV-2-Cre induced GCaMP6s expression in CStr neurons of reporter mice. **b**, GCaMP6s expression throughout brain regions (left) and dorsal cortex (right). Scale bars, 100 µm. **c**, CStr activity during auditory discrimination. Trial averages over all correct, leftward trials in different trial episodes. **d**, Mean activity for auditory (left) and frontal cortex (right) over all CStr (orange) and IT (gray) mice. Dashed lines indicate initiation and response times, gray areas indicate stimulus period, and shading is the s.e.m. *n* = 4 mice per group. **e**, Left, contralateral stimulus kernel, averaged over four CStr mice, 0–200 ms after stimulus onset. Right, traces show changes in auditory (blue), parietal (red) and frontal cortex (yellow). Dashed circles indicate cortical locations in the weight map. **f**, Top, weights from **e** zoomed-in for parietal cortex. Bottom, difference of contralateral versus ipsilateral stimulus kernels. **g**, Left, choice decoder weights during the delay period, averaged over CStr mice. Right, baseline-corrected decoder weights in parietal (left) and frontal (right) cortcices for CStr (orange) and IT mice (gray). Traces were realigned to the initiation, stimulus, delay and response periods (gaps in traces). **h**, Weights from **g** in frontal cortex. **i**, Two-photon field-of-view images in Camk2α-tTA;G6s2 mice with GCaMP6s expression in all PyNs (green) and retrograde-labeled CStr neurons (red). **j**, Left, choice-tuned neurons in ALM (top) and MM (bottom; cortical depth of 200–400 µm). AUC values below 0.5 indicate stronger responses for ipsilateral choices. Right, trial-averaged activity of choice-selective neurons for ipsilateral (red) versus contralateral choices (blue). CStr neurons in ALM (top right) show higher activity for ipsilateral choices. **k**, Fraction of ipsilateral versus contralateral choice-selective cells. Top, more CStr neurons in ALM were ipsi-selective (CStr_Ipsi_, 20.4%; CStr_Contra_, 15.5%; *P* = 0.0018, *n* = 450 cells), while more unlabeled neurons were contra-selective (unlabeled_Ipsi_, 14.3%; unlabeled_Contra_, 17.2%, *P* = 3.5 × 10^−10^, *n* = 4,179 cells). Bottom, most CStr and unlabeled neurons in the MM were contra-selective (CStr_Ipsi_, 10.2%; CStr_Contra_, 19.1%; *P* = 2.7 × 10^−8^, *n* = 315 cells; unlabeled_Ipsi_, 9.3%; unlabeled_Contra_, 19.6%; *P* < 1 × 10^−10^, *n* = 3,450 cells). Data are presented as the mean ± 95% confidence intervals. Asterisks indicate Bonferroni-corrected *P* < 0.01, two-sided binomial test.

The decoder recovered fine-structured choice maps, especially in frontal cortex, revealing contralateral and ipsilateral choice signals in PT and IT mice, respectively. This unexpected inversion could be due to different choice selectivity of specific IT subtypes: intracortical versus CStr projection neurons. Earlier work suggested an even distribution of ipsilateral and contralateral choice selectivity in frontal intracortical projection neurons^20,21^. We thus hypothesized that IT choice selectivity is shaped by CStr neurons. To address this, we developed a retrograde labeling approach by injecting CAV-2-Cre in reporter mice to induce widespread expression of GCaMP6s in CStr neurons (Fig. 7a,b).

**Extended Data Fig. 8:**
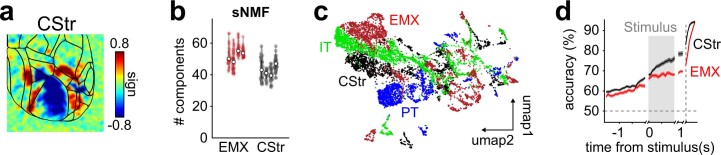
Retrograde labeling of CStr neurons reveals distinct cortical dynamics. (**a**) Visual sign maps from retinotopic mapping experiments. CStr neurons responded to visual stimulation and reveal comparable retinotopic organization as other PyN types. (**b**) Number of sNMF components, accounting for 99% of cortical variance in EMX and CStr mice, dots represent individual sessions. CStr neurons required less components as EMX and IT but more components as PT neurons (compare with Fig. 2a). (**c**) UMAP embedding of spatial sNMF components for EMX (red), IT (green), PT (blue) and CSt (black) mice. Dots show individual spatial components. CStr components were clearly distinct from other PyN types. (**d**) Cross-validated choice-decoder accuracy. Results are shown for EMX (red) and CStr mice (black). Decoder accuracy continuously increased throughout the trial for all PyN types. Dashed line indicates time of response, gray area is the stimulus period.

Using widefield imaging, we observed robust CStr-related fluorescence (Supplementary Video 5) and identified visual areas using retinotopic mapping (Extended Data Fig. 8a). sNMF showed that the dimensionality of CStr mice was intermediate between PT and IT activity, with spatial components forming independent clusters from other PyN types (Extended Data Fig. 8b,c). The clear difference between IT and CStr mice suggests that IT dynamics represent a mixture of intracortical-projecting and corticostriatal-projecting IT neurons with distinct activity patterns.

Trial-averaged CStr activity during the auditory task partially resembled IT activity (for example, in frontal cortex) but also showed clear differences, such as a lack of pre-stimulus suppression in sensory cortex (Fig. 7c,d). CStr and IT mice also differed in their respective stimulus kernels: stimulus-related CStr activity in parietal cortex was stronger than in sensory and frontal cortex but the peak parietal activity was more medial compared to IT mice (Fig. 7e). Interestingly, the location of stimulus-driven parietal regions (Fig. 7f) closely resembled the anatomical and functional topography to the dorsomedial striatum^44,45^. As with PT neurons (Fig. 5b,d), parietal CStr responses were similar for contralateral and ipsilateral stimulation (Fig. 7f).

To determine if CStr activity contributed to ipsilateral-preferring IT choice signals, we used the decoder that predicted choices with equally high accuracy as for PT and IT mice (Extended Data Fig. 8d). We then extracted choice weights for each task episode. CStr activity was overall similar to IT mice, with an even stronger ipsilateral choice preference in frontal cortex that started after stimulus onset and lasted throughout the delay and response periods (Fig. 7g). This inversion from contralateral to ipsilateral choice preference was again prominent in ALM but did not extend to MM, strongly suggesting that ipsilateral choice preference is driven by IT-CStr neurons.

**Extended Data Fig. 9:**
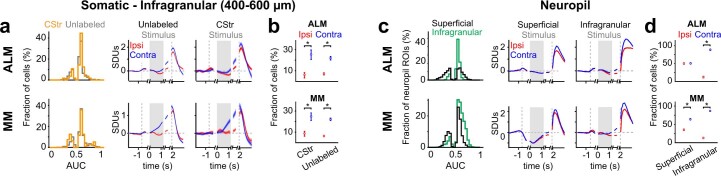
Infragranular CStr neurons are tuned to contralateral choices. (**a**) Left: Overview of significantly choice-tuned neurons in deeper layers (400–600 μm) of ALM (top) and MM (bottom). Orange line: CStr neurons, labeled by tdTomato. Gray lines: unlabeled PyNs. AUC values below 0.5 indicate stronger responses for ipsilateral choices. Right: Trial-averaged activity for all choice-selective neurons, separated for ipsi- (red) versus contralateral choices (blue). Both CStr and unlabeled neurons show strong contralateral choice tuning with no clear difference between PyN-types (*p* < 1 × 10^−10^ for all conditions). This suggest that ipsilateral choice tuning is limited to IT-CStr neurons in superficial layers of ALM. (**b**) Fraction of cells responding selectively for ipsi- (red) versus contralateral choices (blue) in ALM and MM. CStr and unlabeled neurons in both show similar contralateral choice tuning. (ALM: n_CStr_ = 177 cells, n_Unlabeled_ = 1190 cells; MM: n_CStr_ = 287 cells, n_Unlabeled_ = 2620 cells). (**c**) To differentiate somatic versus neuropil choice signals, we quantified ipsi- and contralateral choice tuning for neuropil ROIs. Each neuropil ROI represents the background fluorescence that surrounded a given somatic ROI. Conventions as in a). (**d**) Fraction of neuropil ROIs responding selectively for ipsi- versus contralateral choices. Conventions as in (**b**). Neuropil ROIs were equally tuned to ipsi- and contralateral choices in superficial ALM layers (*p* = 0.14) but otherwise showed contralateral choice-specificity (*p* < 1 × 10^−10^ for other conditions), generally recapitulating choice-specificity from unlabeled neurons. The symmetry found in neuropil choice tuning might explain the bilateral ALM activation observed with IT-specific widefield imaging (Fig. 3c), suggesting that IT-specific widefield signals are comprised of somatic and neuropil activity in the superficial cortex. In contrast, the stronger choice-selectivity in EMX- and PT-specific widefield imaging, suggests that these signals may emerge from infragranular neural activity . (ALM: n_CStr_ = 177 cells, n_Unlabeled_ = 1190 cells; MM: n_CStr_ = 287 cells, n_Unlabeled_ = 2620 cells). Data are presented as mean +/− 95% confidence intervals. Stars indicate bonferroni-corrected p < 0.01, all tests are two-sided binomial test.

To confirm these results at cellular resolution, we recorded all PyNs in frontal cortex with two-photon calcium imaging and identified CStr neurons through retrograde viral labeling (Fig. 7i). Comparing the choice tuning of CStr and unlabeled PyNs revealed a specific difference in ipsilateral versus contralateral choice preference in the ALM (Fig. 7j). Most choice-selective CStr neurons preferred ipsilateral choices, whereas unlabeled PyNs were mildly contra-selective (Fig. 7k). In agreement with our widefield results, these differences were seen in the ALM but not the MM. Interestingly, ipsilateral choice preference was restricted to superficial IT-CStr neurons (cortical depth, 200–400 µm). Infragranular CStr neurons (400–600 µm), which are also often PT cells^17^, showed strong contralateral choice tuning (Extended Data Fig. 9a). Lastly, we tested if neuropil choice signals may have masked somatic activity in our widefield measures. Neuropil largely resembled somatic choice tuning of unlabeled neurons (Extended Data Fig. 9b), confirming that PyN-type-specific widefield measures indeed represented local somatic activity. Here, IT-specific widefield signals matched the mixed choice-selectivity of superficial layers, while PT-specific imaging was well aligned with the clear contralateral choice tuning in deeper cortical layers.

### Pyramidal neuron-type-specific causal contributions to perception and choice formation

**Fig. 8:**
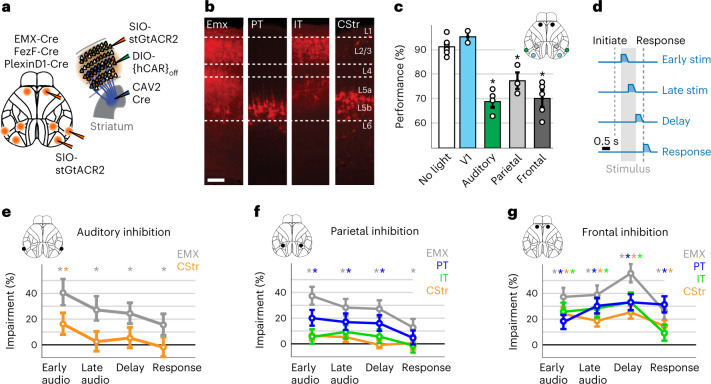
Temporally restricted, pyramidal neuron-specific inactivation of parietal and frontal cortex disrupts decisions. **a**, Left, schematic of injection scheme to induce stGtACR2 expression in EMX, IT or PT neurons. V1 injections were performed in a subset of EMX mice. Right, intersectional viral approach for targeting CStr neurons. A mixture of AAV-DJ-hSYN-DIO-{hCAR}_off_ and AAV1-SIO-hSyn1-stGtACR2-FusionRed was injected into the cortex to enhance CAV-2-Cre uptake, subsequently inducing stGtACR2 expression in CStr neurons. **b**, Laminar distribution of stGtACR2-FusionRed in EMX, PT, IT and CStr neurons. **c**, Behavioral performance (percentage correct) of EMX mice during inactivation of V1 (*n* = 2 mice), auditory (*n* = 5 mice), parietal (*n* = 3 mice) or frontal (*n* = 5 mice) cortex. Data are presented as the mean ± s.e.m. Circles denote individual mice. **d**, Schematic of optogenetic inactivation paradigm; 0.5-s-long optogenetic inhibition was performed during the first or last half of the stimulus period, the subsequent delay or the response period. Light power ramped down after 0.3 s. **e**, Behavioral impairment (percentage change from control performance) with inhibition of EMX or CStr neurons in auditory cortex. Circles denote mean impairments, and error bars represent 95% confidence intervals. *n*_EMX_ = 702, *n*_CStr_ = 834 trials. **f**, Behavioral impairments from parietal PyN-type-specific inhibition. Conventions as in **e**. *n*_EMX_ = 1,627, *n*_PT_ = 1,082, *n*_IT_ = 890, *n*_CStr_ = 1,033 trials. **g**, Behavioral impairments from parietal PyN-type-specific inhibition. *n*_EMX_ = 1,888, *n*_PT_ = 1,304, *n*_IT_ = 791, *n*_CStr_ = 1,372 trials. Conventions as in **f**. Asterisks indicate Bonferroni-corrected *P* < 0.01, two-sided binomial test.

The observed differences between PyNs suggest that each type may drive distinct aspects of decision-making. To causally test their functional role, we performed PyN-type-specific optogenetic inactivation in auditory, parietal and frontal cortex, using the inhibitory opsin stGtACR2 (ref. ^46^; Fig. 8a,b). For CStr neurons, we used an intersectional approach to maximize the efficiency of retrograde expression and reduce potential viral tropism^47^. Cortical inactivation coordinates were determined from our stimulus and choice analyses (Figs. 5a and 6a). To test whether optogenetic effects are area specific, we also targeted the primary visual cortex (V1) in a subset of EMX mice.

As expected, decision accuracy was impaired by bilateral silencing of EMX neurons in auditory, parietal or frontal cortex but unaffected by silencing V1 (Fig. 8c). We then inactivated each area for 0.5 s during four different task episodes: early and late stimulus (the first and last 0.5 s of the stimulus), delay and response (Fig. 8d). Consistent with the notion that auditory and parietal cortex reflect stimulus-driven activity (Fig. 5a), silencing either area strongly impaired task performance, particularly during the stimulus period (Fig. 8e,f). Behavioral impairments (the normalized difference between performance in non-optogenetic trials and chance) were weaker during the subsequent periods, indicating that these areas are most important for early processing of auditory stimuli.

Consistent with earlier work^48^, silencing CStr neurons in A1 impaired auditory decisions (Fig. 8e). However, the effects were more transient and weaker compared to silencing EMX neurons, suggesting that CStr neurons were not exclusively required for accurate task performance. Inactivating IT or CStr neurons in parietal cortex caused surprisingly mild effects, while silencing PT neurons robustly impaired performance (Fig. 8f). This indicates that subcortical PT projection from parietal cortex are more important for sensory processing than intracortical IT or CStr projections, suggesting a role for PT neurons beyond movement preparation and execution.

**Extended Data Fig. 10:**
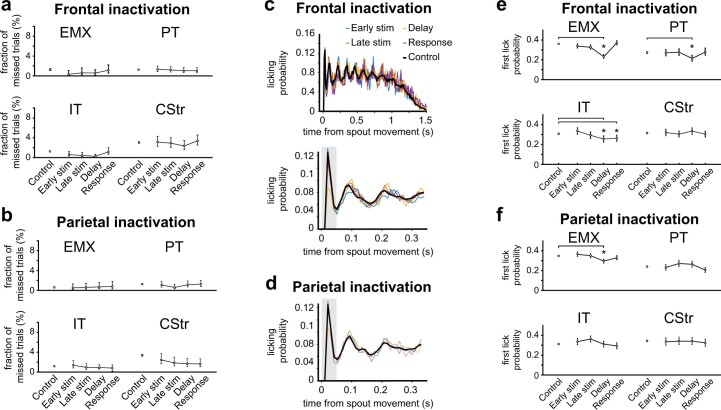
Effects of optogenetic perturbation in frontal and parietal cortex on licking behavior. (**a**) To test if frontal or parietal inactivation impairs movement planning or execution, we computed the fraction of missed trials with and without optogenetic inhibition in frontal cortex. No increase in missed trials was detected, demonstrating that the animals’ ability to respond was not impaired (n_EMX_ = 2009, n_PT_ = 1192, n_IT_ = 669, n_CStr_ = 1408 trials). (**b**) Same as in a) for parietal inactivation (n_EMX_ = 1721, n_PT_ = 1108, n_IT_ = 844, n_CStr_ = 1093 trials). (**c**) Top: Quantification of licking behavior after spouts were moved in for all correct trials in a single EMX animal (10 ms bins). Licking probability varies rhythmically at ~10 Hz as the animal licks the spout repeatedly (black line). The same pattern is observed with frontal optogenetic inactivation in different trial episodes (colored lines), demonstrating that motor generation is not generally perturbed. Bottom: While the lick pattern is largely similar with optogenetics, inactivation during the delay period (yellow line) reduces the lick probability during the first 40 ms (gray area). Frontal inactivation during the delay might thus increase animals’ reaction times. (**d**) Same as in (**c**) but for parietal inactivation. (**e**) Quantification of lick probability in the first 40 ms for all cell types. Frontal inactivation during the delay period reduces early lick probability in EMX, PT and IT mice but not CStr mice (n_EMX_ = 2009, n_PT_ = 1192, n_IT_ = 669, n_CStr_ = 1408 trials). (**f**) Same as in (**e**) for parietal inactivation. Only EMX inactivation during the delay caused a small reduction in first lick probability (n_EMX_ = 1721, n_PT_ = 1108, n_IT_ = 844, n_CStr_ = 1093 trials). Data are presented as mean +/− 95% confidence intervals. Stars indicate bonferroni-corrected *p* < 0.005, two-sided binomial test.

Frontal inactivations resulted in the strongest impairment, with IT and CStr inactivation causing similar effects during the stimulus and delay periods (Fig. 8g). Impairments in IT mice are therefore not solely due to the disruption of intracortical processing^20^ but also involve alterations of CStr neurons. Inactivating PT neurons equally impaired performance during the stimulus and delay period but showed stronger effects during the final response period. Impairments in the response period were similar for EMX and PT mice, suggesting that PT neurons are particularly involved in licking responses. Multiple PyN types in frontal cortex are therefore involved in the formation and maintenance of choices, despite clear differences in their respective choice tuning. Lastly, we also analyzed licking patterns to test if optogenetic inhibition broadly disrupted animal movements. Frontal inactivation in the delay period had a mild effect on response latency but did not affect response probability or licking patterns, arguing against a strong motor impairment (Extended Data Fig. 10). PyN-type-specific inhibition therefore selectively reduced the animals’ response accuracy rather than broadly disrupting their ability for movement initiation and execution.

## Discussion

We measured and manipulated PyN types to determine whether they play distinct roles in decision-making. Cortex-wide activity patterns were PyN-type specific, each reflecting distinct neural dynamics at multiple spatial scales. Functional specificity across PyN types was also evident during decision-making: each PyN type exhibited unique cortical localization and specificity associated with stimulus and choice. These response patterns were not seen when imaging from PyNs nonspecifically. PyN-type-specific optogenetic inactivation confirmed distinct functional roles in parietal and frontal cortex, highlighting the importance of subcortical projections for decision formation. Our results suggest that different PyN types are functionally distinct, and perform separate roles during auditory decision-making.

Dimensionality reduction of cortical dynamics^33,49,50^ revealed that nearly all spatial components were PyN-type specific. Large-scale activity patterns are therefore shaped by PyN-specific dynamics. This has important implications for studies of cortex-wide neural dynamics, which are often based on nonspecific measures of neural activity^51–54^. Earlier work revealed functional modules that span the entire cortex^49,53–56^ and follow intracortical connectivity patterns^57,58^. Our results point to the existence of additional, PyN-type-specific motifs, especially for subcortical projections, such as PT or CStr neurons. Furthermore, most PyN-specific LocaNMF components consisted of spatially precise subregions that were smaller than classic cortical areas. Future studies could reveal even more detailed cortical structures by combining large-scale measures of multiple PyN types with multicolor widefield imaging^24,59^ and observing interactions between PyN-specific cortical dynamics within the same animal.

We also observed unique sensory response patterns for each PyN type. This is in line with recent results from primary somatosensory^18,60^ and visual cortex^19^, arguing that different PyN types play separate roles during sensory processing. The clear differences in magnitude, localization and lateralization of sensory responses in parietal and frontal cortex demonstrate that the functional specialization of different PyN types is a general feature of cortical circuit function.

Correspondingly, we found diverse behavioral effects when inactivating PyN types. Consistent with earlier work, inactivating CStr neurons in auditory cortex impaired task performance^48^, suggesting that corticostriatal projections are important for sensory perception. Inactivating parietal cortex also caused strong behavioral impairments during sensory stimulation but not when silencing CStr or IT neurons. This shows that the importance of CStr projections does not generalize from auditory to parietal cortex and also argues against models in which sensory information is intracortically transmitted from parietal to frontal cortex during decision formation^42,61,62^. Instead, silencing parietal PT neurons during the stimulus presentation strongly disrupted decisions, highlighting the importance of subcortical projections for decision-making^63^.

These results are at odds with earlier studies in rats, showing little or no impact of parietal inactivation on auditory performance^64,65^. Conversely, other work in head-fixed mice reported robust impairments in visual^43,66–68^ and auditory^62^ tasks. This could be due to differences between rats and mice or the precise location of parietal inactivation. Sensory modalities are processed along a mediolateral gradient in parietal cortex, emphasizing the need to precisely target specific parietal areas to obtain a modality-specific behavioral effect^62^. Our task also requires evidence accumulation and working memory, which engage a wider range of cortical regions and could explain the involvement of parietal cortex for accurate decisions^67^.

The accumulation and memory requirements might also explain why we found clear cortical choice signals, whereas recent cortex-wide studies reported little choice selectivity^39,69^. The lack of side-specific choice tuning in IT populations matches earlier work, showing that intracortical projections in ALM equally include contralateral and ipsilateral choice-preferring cells^20,21^. In contrast, CStr populations were more selective for ipsilateral choices and we confirmed that this was also present in individual CStr neurons. PyN-specific widefield signals therefore selectively reflect somatic activity and not just superficial neuropil signals. Ipsilateral choice signals in CStr neurons were restricted to superficial ALM, which is mostly implicated in movement generation^13,70^. A recent study showed that CStr projections from the anterior cingulate cortex inhibit striatal activity and motor behavior^71^. Ipsilateral choice tuning of CStr neurons could therefore serve to disinhibit striatal circuits when releasing a targeted licking response.

Frontal inactivation strongly impaired animal behavior during the stimulus and delay periods, suggesting an important role for the translation of sensory inputs into behavior^21,69,72–74^. Impairments were largely similar across frontal PyN types, which appear to be equally required for choice formation and retention. Frontal PyNs may thus be more reliant on each other to maintain accurate function than in sensory areas^18,19,48^. As the only exception, PT neurons were more important during the response period, consistent with a specific role of brainstem-projecting PT neurons for motor execution^13^.

Our work offers a new perspective on cortex-wide dynamics by viewing them through the lens of different PyN types and strongly supports the view that cortical circuits perform parallel computations, even within the same cortical layer^13,18,19,75^. Future work to reveal how cortical circuits generate behavior should therefore include PyN types to resolve the heterogeneity that is often encountered when studying cortical decision circuits. A powerful tool to achieve this goal are novel mouse lines, such as inducible knock-in lines that permit reliable targeting of PT and IT neurons. These mouse lines also overcome several earlier problems, such as unstable expression patterns or cell-type mixtures due to interactions with surrounding genetic elements^26,76^. Moreover, combining genetic mouse lines with retrograde labeling will enable the targeting of specific PyN subtypes, such as projection-specific PT neurons^12,13^, that might serve a large array of functions from sensory processing, to working memory and motor function^13,18^.

## Methods

### Mouse lines

All surgical and behavioral procedures conformed to the guidelines established by the National Institutes of Health (NIH) and were approved by the Institutional Animal Care and Use Committee of Cold Spring Harbor Laboratory. Mice were 8- to 25-week-old males (Supplementary Table 2). No statistical methods were used to predetermine sample sizes but sample sizes are similar to those reported in previous publications^22,25^. Mouse strains were acquired from the Jackson Laboratory, Allen Brain Institute, or generated at Cold Spring Harbor Laboratory. The mouse room had a relative humidity of 30–70%, and a room temperature of 69–78 °F. Transgenic strains crossed to generate double- and triple-transgenic mice used for imaging were: Emx-Cre (JAX 005628), LSL-tTA (JAX 008600), Ai93D (JAX 024103), Ai162 (JAX 031562), TRE-GCaMP6s (G6s2, JAX 024742) and H2B-eGFP (JAX 006069; Supplementary Table 3). EMX mice, used for calcium imaging, were bred as Ai93D;Emx-Cre;LSL-tTA. To avoid potential aberrant cortical activity patterns, EMX mice were on a doxycycline (DOX)-containing diet, preventing GCaMP6s expression until they were 6 weeks or older^22,25^.

For widefield imaging of PT and IT neurons, inducible knock-in drivers Fezf2-2A-CreER and PlexinD1-2A-CreER, respectively, were crossed with Ai162 reporter mice to drive cortex-wide GCaMP6s expression. Cre expression was induced through two doses of intraperitoneal injections of tamoxifen (200 mg per kg body weight; 20 mg ml^−1^ corn oil solution) at postnatal day (P) 28 and P32, yielding expression patterns consistent with prior reports^26^. For widefield imaging of corticostriatal neurons, we crossed Ai162 with G6s2 to create a double-transgenic reporter strain Ai162;G6s2 with two hemizygous copies of GCaMP6s under tetO control. Because LSL-tTA is incorporated in tandem with the reporter gene in the Ai162 strain^29^, this hybrid reporter line permits Cre-dependent expression of GCaMP6s at higher levels than Ai162 hemizygotes while avoiding potential leaky reporter gene expression. To achieve widespread GCaMP6s expression in corticostriatal neurons, we performed striatal injections of retrograde virus (CAV-2-Cre) in the hybrid Ai162;G6s2 reporter line (see ‘Viral injections’). For two-photon imaging, GCaMP6s expression in PyNs was generated using the hybrid strain Camk2α-tTA;G6s2.

### General surgical procedures

Surgeries were performed under 1–2% isoflurane in oxygen anesthesia. After induction of anesthesia, 1.2 mg per kg body weight meloxicam was injected subcutaneously and sterile lidocaine ointment was applied topically to the skin incision site. After making a midline cranial incision, the skin was retracted laterally and fixed in position with tissue adhesive (Vetbond, 3M). We then built an outer wall using dental cement (C&B Metabond, Parkell; Ortho-Jet, Lang Dental) along the lateral edge of the dorsal cranium (frontal and parietal bones). A custom titanium skull post was then attached to the dental cement. For skull clearing, the skull was thoroughly cleaned followed by the application of a thin layer of cyanoacrylate (Zap-A-Gap CA+, Pacer technology)^23^.

For two-photon imaging, a circular craniotomy (*ø* = 3 mm) over the right frontal cortex (1.75 mm lateral and 1.75 mm rostral to bregma), was made using a biopsy punch. A circular coverslip (*ø* = 3 mm) was then lowered to the surface of the brain and sealed to the skill with Vetbond and Metabond. Lastly, a titanium skull post was implanted as described above.

### Viral injections

After induction with isoflurane anesthesia, animals were placed in a stereotaxic frame (David Kopf Instruments). Injections were made using a programmable nanoliter injector (Nanoject III, Drummond Scientific). For widefield imaging of CStr mice, widespread corticostriatal GCaMP6s expression was generated in Ai162;G6s2 reporter mice by performing bilateral stereotaxic injections of CAV-2-Cre (at 3–4 weeks of age) into the dorsal striatum at three targets per hemisphere, spanning the rostrocaudal (RC) axis. The target coordinates (relative to bregma and dura, in mm) are: (1) RC + 0.75, mediolateral (ML) ± 1.8, dorsoventral (DV) 3.0; (2) RC 0, ML ± 2.2, DV 3.1; (3) RC −0.75, ML ± 2.9, DV 3.1. For each striatal target, a burr hole was created using a small dental burr followed by injection of 1.8 × 10^9^ purified particles (pp) of CAV-2-Cre using pipettes with long taper tips pulled from borosilicate capillaries (3.5 inch, 3-000-203-G/X, Drummond Scientific). For two-photon imaging experiments, CStr neurons were labeled through striatal injections of AAV-2-retro-CAG-tdTomato (using the same approach and coordinates as described above) in Camk2α-tTA;G6s2 mice.

For cell-type-specific optogenetic silencing experiments, we performed bilateral injections in frontal, parietal and auditory cortices (coordinates relative to bregma: frontal: RC + 2.5 mm, ML ± 1.5 mm; parietal: RC −1.7 mm, ML ± 2.5 mm; auditory: RC −2.5 mm, ML ± 4.6 mm) to induce expression of Cre-dependent stGtACR2 (AAV1-hSyn-SIO-stGtACR2-FusionRed, Upenn Vector Core). Cortical injections were performed in P42 to P56 Fezf2-2A-CreER, PlexinD1-2A-CreER and EMX-Cre reporter mice. In CreER mice, intraperitoneal tamoxifen was administered 1 week after viral injections. Cortical injections were made at 300 and 600 µm per area. In two EMX-Cre mice, bilateral injections were performed in the frontal and visual cortex (RC −4, ML ± 2.5). To target CStr neurons, injections were performed in C57BL/6J mice in two stages. First, we utilized a viral receptor complementation strategy^47^ by injecting both AAV-DJ-hSYN-DIO-{hCAR}_off_ and AAV1-SIO-hSyn1-stGtACR2-FusionRed (Supplementary Table 3) in cortex (coordinates as described above) in P21–P28 mice. Second, we performed bilateral striatal CAV-2-Cre injections, 6 weeks after cortical injections. hCAR is expressed in all transfected neurons in a Cre-OFF manner, where Cre expression stops expression of hCAR while simultaneously inducing stGtACR2 expression.

### Optical fiber implantation

For optogenetic silencing, we used the soma-targeted anion-conducting channelrhodopsin stGtACR2 (ref. ^46^). Optical fibers (NA = 0.36, *ø* = 0.4 mm, FT400UMT, Thorlabs) were glued into metal or ceramic ferrules (*ø* = 1.25 mm, Thorlabs) and secured above the cortex following viral injections. Ferrule-enclosed optical fiber implantations immediately followed cortical AAV (Supplementary Table 3) injections in Fezf2, PlexinD1 and Emx mice and striatal injections in CStr mice. One polished end of the optical fiber was positioned extradural to the site of cortical injections and interfaced with thinned skull using cyanoacrylate. Next, the fiber was fixed to the skull using light-cured glass ionomer (Vitrebond, 3M). Additional layers of dental cement and dental acrylic (Lang Dental Jet Repair Acrylic, 1223MEH) were applied around the fiber implant and the skull to reinforce for durability and long-term stability. After all layers were cured, a final outer coating of cyanoacrylate and nail polish were applied.

### Behavioral training

The behavioral setup was controlled with a microcontroller-based (Arduino Due) finite state machine (Bpod r0.5, Sanworks) using custom MATLAB code (2015b, MathWorks) running on a Linux PC. Servo motors (Turnigy TGY-306G-HV) and touch sensors were controlled by microcontrollers (Teensy 3.2, PJRC) running custom code. Fifty-four mice were trained on a delayed, spatial discrimination task. Mice initiated trials by placing their forepaws on at least one of the two handles, which were mounted on servo motors that rotated out of reach during the intertrial period. Upon trial initiation, animals placed their forepaws on the handles and, after a variable duration of 0.25–0.75 s of continuous contact, the auditory stimulus was presented. Auditory stimuli consisted of a sequence of Poisson-distributed, 3-ms-long auditory click sounds^36^, presented from either a left and/or a right speaker for a variable duration between 1 and 1.5 s. The stimulus period was followed by a variable delay of up to 1 s, then the servo motors moved two lick spouts close to the animal’s mouth. If the animal licked twice on the side where more clicks were presented, a drop of water reward was dispensed. The amount of water rewarded for each trial (typically 1.5 to 3 µl) was constant within a single session but was sometimes adjusted daily based on the animal’s body weight. After a spout was licked twice, the contralateral spout moved out of reach to force the animal to commit to its decision.

All trained mice were housed in groups of two or more under a reverse light cycle (12-h dark and 12-h light) and trained during their active dark cycle. Animals were trained over the course of approximately 30–60 d. After 2–3 d of restricted water access, animals began habituation to head fixation and received water from spouts in the behavior chamber. During these sessions, unilateral auditory stimuli were presented followed by a droplet of water from the ipsilateral water spout. After several habituation sessions, animals were required to touch the handles to trigger stimulus presentation. Once mice could reliably reach for the handles, the required touch duration was progressively increased to 0.75 s. During the next training stage, both spouts moved within reach of the animal following stimulus presentation. An animal was considered trained when its detection performance across two or more sessions was >80%.

### Behavioral monitoring

Data were collected from multiple sensors in the behavioral setup. Touch sensors using a grounding circuit on handles and lick spouts detected contact with the animal’s forepaws and tongue, respectively. A piezo sensor (1740, Adafruit) below the animal’s trunk was used for monitoring body and hindlimb movements. Two webcams (C920 and B920, Logitech) were positioned to capture the animal’s face (side view) and the ventral surface of the body (ventral view).

### Widefield imaging

Widefield imaging was done as reported previously^23,32,77^ using an inverted tandem-lens macroscope and an sCMOS camera (Edge 5.5, PCO) running at 30 frames per second (fps). The focal lengths of the top lens (DC-Nikkor, Nikon) and bottom lens (85M-S, Rokinon) were 105 mm and 85 mm, respectively. The field of view was 12.5 × 10.5 mm^2^ and the imaging resolution was 640 × 540 pixels after 4× spatial binning, resulting in a spatial resolution of ~20 μm per pixel. To capture GCaMP fluorescence, a 525-nm bandpass filter (86-963, Edmund optics) was placed in front of the camera. Using excitation light at two different wavelengths, we isolated Ca^2+^-dependent fluorescence and corrected for intrinsic signals (for example, hemodynamic responses)^22,25^. Excitation light was projected on the cortical surface using a 495 nm long-pass dichroic mirror (T495lpxr, Chroma) placed between the two macro lenses. The excitation light was generated by a collimated blue LED (470 nm, M470L3, Thorlabs) and a collimated violet LED (405 nm, M405L3, Thorlabs) that were coupled into the same excitation path using a dichroic mirror (87-063, Edmund optics). We alternated illumination between the two LEDs from frame to frame, resulting in one set of frames with blue and the other with violet excitation at 15 fps each. Excitation of GCaMP at 405 nm results in non-calcium-dependent fluorescence^78^, allowing us to isolate the true calcium-dependent signal by rescaling and subtracting frames with violet illumination from the preceding frames with blue illumination. Subsequent analyses were based on this differential signal. Imaging data were then rigidly aligned to the Allen Mouse Brain Common Coordinate Framework (CCF), using four anatomical landmarks: the left, center and right points where anterior cortex meets the olfactory bulbs, and the medial point at the base of retrosplenial cortex. Retinotopic visual mapping experiments^30,79^ confirmed accurate CCF alignment and showed high correspondence between functionally identified visual areas and the CCF across PyN types (Fig. 1c).

### Two-photon imaging

We used a two-photon resonant scanning microscope (Moveable Objective Microscope, Sutter Instruments) for continuous image acquisition at 30.9 Hz. A ×16, 0.8-NA Nikon objective lens was used for single-plane imaging with a field of view of 512 × 512 pixels (575 µm × 575 µm). Mode-locked illumination at 930 nm was delivered using a Ti:Sapphire laser (Ultra II, Coherent). The depth of focal planes was 200–600 µm below the dura. Emission was collected using bandpass red (670/50 nm) and green (525/50 nm) filters (Chroma Technologies). MScan software (Sutter Instruments) was used for image acquisition. Recordings were performed in ALM (2.5 mm rostral and 1.5 mm lateral to bregma) or MM (1.5 mm anterior and 1 mm lateral to bregma) in randomized order across mice. Across imaging session, we selected planes that differed from those of prior sessions to maximize the number of unique neurons.

Raw images were processed using the Suite2P package^80^ to perform motion correction, model-based region of interest (ROI) detection, correction for neuropil contamination and spike deconvolution. Somatic and non-somatic (neuropil) ROI identification was performed through a combination of a pretrained classifier and manual curation. Somata with tdTomato expression were identified in a two-step process. First, potential green channel bleed-through was subtracted from the red channel using nonrigid regression with individual channels being divided into smaller blocks. Next, all sessions were manually inspected to identify a conservative red fluorescence threshold, which was subsequently applied to all sessions. Analyses of neural activity were based on deconvolved values (‘inferred spiking activity’). Because the deconvolved values do not represent absolute firing rates, we performed *z*-score normalization for each neuron before computing trial averages across cells. The total number of recorded neurons for each session was 396 ± 105 (mean ± s.d.).

### Optogenetic inactivation

Photostimulation was performed using a 470-nm high-power LED (M470F3, Thorlabs) with a power density of 25 mW/mm^2^. Stimuli consisted of a square-wave stimulus that ramped down in power for 200 ms, to avoid an excitatory post-illumination rebound due to sudden release of inhibition^81^. To prevent animals’ visual detection of photostimulation, through either external leakage from light-insulated mating sleeves or transmission to the retina across the brain, an external LED with matching wavelength placed at the center of the animal’s visual field was flashed throughout the duration of every trial. Photoinhibition was performed in 20% of total trials and randomly interleaved between light-off trials. Once an animal was habituated and able to complete detection behavior trials with >90% accuracy, bilateral optogenetic inactivation trials were introduced. During these initial sessions, optogenetic inhibition was performed from the beginning of the stimulus epoch until the end of the delay epoch. Additionally, we performed 0.5-s inhibition during four predefined epochs of the detection behavior trials: (1) first half of the stimulus, (2) second half of the stimulus, (3) delay and (4) response.

### Immunohistology, microscopy and image analysis

After behavioral experiments, we performed transcardial perfusion with PBS followed by fixation with 4% paraformaldehyde in 0.1 M phosphate buffer. Brains were post-fixed in 4% paraformaldehyde for an additional 12–18 h at 4° C. Before sectioning, brains were rinsed three times in PBS and embedded in 4% agarose-PBS. Then, 50-μm-thick slices were made using a vibrating microtome (Leica, VT100S). Sections were then suspended in blocking solution (10% Normal Goat Serum and 0.1% Triton-X100 in 1× PBS) for 1 h followed by overnight incubation at 4 °C with the primary antibody. Next, sections were washed with PBS, incubated for 1 h at room temperature with the secondary antibody at 1:500 dilution. For histological visualization of GCaMP6s, we used primary goat polyclonal anti-GFP antibody (1:500 dilution; Abcam, ab6673) and secondary donkey anti-goat Alexa Fluor 488 (1:500 dilution; Abcam, ab150129). Sections were then dry-mounted on slides using Vectashield (Vector Labs, H1000) before imaging. No immunostaining was performed for the visualization of FusionRed or tdTomato. Imaging was performed using an upright fluorescence macroscope (Olympus BX61). Images were acquired using Ocular Scientific Image Acquisition Software (Teledyne Imaging). Visualization and analysis were performed using ImageJ/FIJI software packages.

### Quantification of cortex-wide gene expression

Cell-count quantification was performed using publicly available serial two-photon tomography datasets (http://www.brainimagelibrary.org/)^26^. Cre expression patterns for IT and PT neurons were characterized with data from eight mice, expressing either Cre-dependent GFP (PlexinD1-2A-CreER;Snap25-LSL-2A-EGFP) or tdTomato (Fezf2-2A-CreER;Ai14), respectively. Cell counting was performed via automated soma detection, using a trained convolutional neural network^82^. Datasets were then registered to the Allen CCF v3 using the Elastix toolbox^83^. To obtain the density of Cre-expressing neurons for individual cortical areas, we used the area outlines from the Allen CCF and computed the average sum of detected IT or PT neurons in each area, normalized by its surface area.

### Preprocessing of neural data

We used a rigid-body image registration method implemented in the frequency domain^84^ to align each imaging frame to the median over all frames in the first trial. To reduce the computational cost of subsequent analyses, we then computed the 200 highest-variance components using singular value decomposition (SVD). These components accounted for at least 95% of the total variance in each recording, whereas computing 500 components accounted for little additional variance (~0.15%). SVD reduces the raw imaging data Y to a matrix of ‘spatial components’ U (of size pixels by components), ‘temporal components’ V^T^ (of size components by frames) and singular values S (of size components by components) to scale temporal components to the original data. The resulting decomposition has the form Y = USV^T^. All subsequent analysis in the time domain (such as the encoder and decoder models described below) were performed on the product SV^T^ and the respective results were later multiplied by U, to recover results for the original pixel space. To avoid slow drift in the imaging data, SV^T^ was high-pass filtered above 0.1 Hz using a zero-phase, second-order Butterworth filter.

To compute trial averages and perform choice decoder analysis (see below), imaging data in individual trials were aligned to the four trial periods, each marked by a specific event. This was required because the duration of different trial events was randomized to reduce temporal correlations, for example, between trial initiation, the stimulus presentation and subsequent lick responses. The first period (initiate) was aligned to the time when animal initiated a trial by touching the handles, the second (stimulus) was aligned to the stimulus onset, the third (delay) to the end of the stimulus sequence, and the fourth (response) to the time when spouts were moved in to allow a lick response. After alignment, the total trial duration was 2 s and durations of respective trial episodes were 0.5 s (initiate), 1 s (stimulus), 0.2 s (delay) and 0.3 s (response).

### Spatial clustering and classification

To obtain more interpretable spatial components and assess the dimensionality of cortical activity in different PyN types, we used sNMF. As with SVD, sNMF creates spatial and temporal components for each session but enforces positive spatial components. Temporal components were not enforced to be nonnegative because hemodynamic correction produces temporal dynamics that can be positive or negative, relative to baseline. We used the LocaNMF toolbox^33^ (https://github.com/ikinsella/locaNMF/) to transform the spatial and temporal components U and SV^T^ into corresponding matrices A and C. A is a matrix of nonnegative spatial components (of size pixels by components). C is the corresponding temporal components (of size components by frames). In addition to regular sNMF, the LocaNMF toolbox can be initialized with spatial constraints based on the Allen CCF. To obtain spatially restricted localized LocaNMF components, we constructed a map of larger seed regions by merging areas in the Allen CCF together (Fig. 2e). This region map was then used to enforce that each component in A is sparse outside the boundary of a given region. To obtain dense spatial components, we used a localization threshold of 50%. For sNMF components, we used the LocaNMF toolbox with a single region that spanned the entire cortex to obtain cortex-wide components while ensuring that all other analysis steps were identical for sNMF and LocaNMF components. In both cases, we determined how many components in A and C were needed to explain 99% of the variance of Y (with Y = AC) after the initial SVD.

To compare spatial sNMF and LocaNMF components from different PyN types, we embedded them in a two-dimensional space, using UMAP analysis (Fig. 2c,g). UMAP analysis was performed with the UMAP toolbox^35^ (https://github.com/lmcinnes/umap/). For each recording, the first 20 spatial components in A were downsampled by a factor of 2, smoothed with a two-dimensional Gaussian filter (5 × 5 pixels, 2-pixel standard deviation) and peak normalized. Components from all recordings and animals were then combined into a larger matrix (of size pixels by components). We used UMAP to project pixels into two, maximally separating nonlinear dimensions. Each point in the two-dimensional space (Fig. 2c,g) reflects a single component from one animal in one imaging session. The same approach was used for temporal sNMF and LocaNMF components. Before the UMAP projection, we first computed the trial-averaged and *z*-scored activity of each component to achieve temporal dynamics that are comparable across sessions and individual mice.

To identify PyN types based on individual spatial components (Fig. 2d,h), we performed a separate UMAP analysis for each mouse. Each of these projections excluded all components from the test animal, ensuring that the UMAP projection was not shaped by potential noise patterns or other unknown features of the test components that could affected the classifier result. We then tested the first 20 components of each session of the test animal with 100 repetitions per component. In every repetition, 1,000 components from each PyN type were randomly selected from the pre-computed UMAP space. We assigned the PyN type of the test component based on the identity of its ten nearest neighbors in UMAP space. For LocaNMF components, we performed the same procedure but additionally enforced an equal number of components from each seed region and PyN type. This prevented PyN types with a larger number of components in a region from biasing the classifier result. Classifier accuracy for each session (Fig. 2d,h) was computed as the mean probability over all repetitions to accurately identify the PyN type.

To determine the size of PyN-predictive LocaNMF components, we selected all spatial components that achieved a classification accuracy of 99% or higher (all other components were assigned as nonspecific) and thresholded each component above 0.2 to obtain binary images. The size of each component was then computed as the square root of the sum of all pixels and converted to square millimeters.

### Linear encoding model

The linear encoding model included task-related and movement-related variables (Supplementary Table 1), as described previously^32^. Each variable consisted of multiple regressors that were combined into a larger design matrix. Binary regressors contained a single pulse that signaled the occurrence of specific events, such as the stimulus onset, and additional regression copies that were shifted forward or backward in time to account for changes in cortical activity before or after the respective event. For auditory stimuli, the time-shifted copies spanned all frames from the onset of the auditory sequence until the end of the trial. Individual click sounds were also captured by an additional regressor set that spanned the 2 s after click onset. For licks and whisks, the time-shifted copies spanned the frames from 1 s before until 2 s after each event. For some variables, for example, previous choice, the time-shifted copies spanned the whole trial. Other variables were analog, such as measures from the piezo sensor, pupil diameter and the 200 highest temporal components of video information from both cameras (using SVD as described above). This ensured that the model could account for animal movements and accurately isolate task-related activity. Movement and task variables were additionally decorrelated due to the variable durations of the initiation, stimulus and delay period. The model was fit using ridge regression to allow for similar contributions from different correlated variables. To determine the regularization penalty *λ* for each column of the widefield data, we used marginal maximum likelihood estimation (MLE)^85^. MLE expresses the encoding model as a Bayesian linear model and determines the ridge penalty *λ* by maximizing the marginal likelihood π(D|*λ*) of the model, given data D. This was done iteratively by testing different *λ* values to determine a global minimum for the negative log-likelihood −log π(D|*λ*). The main advantage of this approach is that *λ* can be determined without computationally expensive cross-validation procedures, resulting in a ~50-fold decrease in required compute time on a regular work station. Moreover, the faster MLE approach allows adjusting *λ* values for individual widefield data components, resulting in higher cross-validated explained variance of the encoding model, compared to a regular cross-validation approach.

### Variance analysis

Explained variance (cvR^2^) was obtained using tenfold cross-validation. This was done by fitting the model weights to a continuous 90%-large section of the imaging data and then computing the explained variance in the remaining 10% of the data. The procedure was repeated ten times, while shifting the training and test data to ensure that each part of the recording was used in the test data in one of the folds. To assess unique explained variance by individual variables (ΔR^2^), we created reduced models in which all regressors of a specific variable were shuffled in time. Shuffling of each regressor was done within individual trials to account for a potential impact of very slow temporal correlations due to the kinetics of the calcium indicator. The difference in explained variance between the full and the reduced models yielded the unique contribution ΔR^2^ of that model variable that could not be explained by other variables in the model. The same approach was used to compute unique contributions for groups of variables, that is, ‘movements’ and ‘task’. Here, all variables that corresponded to a given group were shuffled together.

### Decoding model

To predict animal’s left/right choices from widefield data, we trained logistic regression decoders with an L1 penalty on the temporal component matrix SV^T^ in each session. The L1 penalty was defined as the inverse of the number of observations in the test dataset during cross-validation, which yielded a good balance between the cross-validated prediction accuracy of the decoder and the number of nonzero model regressors. When decoding choice, we randomly removed trials until there were equal numbers of correct and incorrect trials where mice chose the left and the right side. By balancing left/right choices and correct/incorrect trials, we ensured that the decoder would not reflect choices due to corresponding sensory information or side biases. The logistic regression model was implemented in MATLAB using the ‘fitclinear’ function and run repeatedly for each time point in individual trials after realigning them to trial periods as described above. In each session, all decoder runs were performed with the same number of trials (at least 250). We used tenfold cross-validation to compute decoder accuracy at each time point. β-weights were averaged from all models created during cross-validation and convolved with the spatial component matrix U to create cortical maps of the choice decoder weights.

### Receiver-operating characteristic analysis

We computed the area under the receiver-operating characteristic curve (AUC) to quantify choice preference of single neurons obtained from two-photon imaging. AUC values were computed by comparing the mean neural activity during the stimulus and delay period in all trials with ipsilateral versus contralateral choices. AUC values denote the specificity of the neural activity to ipsilateral or contralateral choices, with values below 0.5 signifying ipsilateral choice selectivity and AUC values above 0.5 denoting contralateral choice selectivity. To identify statistically significant choice-selective neurons, AUC values were also computed for shuffled trial labels (randomly assigning ipsilateral and contralateral choices across trials) for each neuron. This procedure was repeated 100 times to create a distribution of shuffled AUC values for each neuron. A neuron’s choice selectivity was then deemed significant if the probability of obtaining the actual AUC from the shuffled AUC distribution was less than 0.05.

### Reporting summary

Further information on research design is available in the Nature Portfolio Reporting Summary linked to this article.

## Online content

Any methods, additional references, Nature Portfolio reporting summaries, source data, extended data, supplementary information, acknowledgements, peer review information; details of author contributions and competing interests; and statements of data and code availability are available at 10.1038/s41593-022-01245-9.

## Supplementary information

Supplementary Information Supplementary Tables 1–3

Reporting Summary

Supplementary Data 1 Editorial Assessment Report

Supplementary Video 1 Widefield calcium imaging data during decision-making from an example Ai93D;Emx-Cre;LSL-tTA mouse (GCaMP6s expressed pan-neuronally).

Supplementary Video 2 Widefield calcium imaging data during decision-making from an example Fezf2-CreER mouse (GCaMP6s expressed in PT neurons only).

Supplementary Video 3 Widefield calcium imaging data during decision-making from an example PlexinD1-CreER mouse (GCaMP6s expressed in IT neurons only).

Supplementary Video 4 Reconstruction of IT neural activity using the sNMF components from IT neural activity (left) and the sNMF components from PT neural activity (right).

Supplementary Video 5 Widefield calcium imaging data during decision-making from an example mouse in which GCaMP6s was expressed only in cortical neurons projecting to the striatum (virally targeted).

## Data Availability

The data from this study are available at 10.25452/figshare.plus.21538458. A link to the data repository with a description of the behavioral and imaging data can be found at https://churchlandlab.dgsom.ucla.edu/pages/code/.

## Code availability

The MATLAB and Python code used for the data analysis in this study is available as a public GitHub repository. The link to the repository can be found at https://churchlandlab.dgsom.ucla.edu/pages/code/.
