# Supplementary material for: Pyramidal cell types drive functionally distinct cortical activity patterns during decision-making
Source: Nat Neurosci. 2023 Jan 23;26(3):495–505. doi: 10.1038/s41593-022-01245-9 (PMC9991922; doi:10.1038/s41593-022-01245-9)
Supplement: Supplementary file 1 — Supplementary Tables 1–3 [file 41593_2022_1245_MOESM1_ESM.pdf]

nature neuroscience

Article https://doi.org/10.1038/s41593-022-01245-9

Pyramidal cell types drive functionally distinct cortical activity patterns during decision-making

In the format provided by the authors and unedited

Table 1. Overview of different model variables

Variable name	Description	Variable type	Category
Hindlimb	Piezo sensor below the animal	Analog + Event kernel	Movement
Handles (Left / Right)	Touch events from handle sensors	Event kernel	Movement
Licks (Left / Right)	Lick events from spout sensors	Event kernel	Movement
Pupil	Pupil diameter, extracted from face camera	Analog + Event kernel	Movement
Nose	Nose movements, extracted from face camera	Analog + Event kernel	Movement
Whisking	Whisker movements, extracted from face camera	Analog + Event kernel	Movement
Body	Average motion energy across all body camera pixels	Analog + Event kernel	Movement
Video	Video dimensions from both cameras (SVD)	Analog	Movement
Video ME	Video dimensions from motion energy in both cameras	Analog	Movement
Choice (Left / Right)	All frames in either a left- or a rightward choice trial	Event kernel	Task
Previous choice	Every trial after a leftward choice trial	Event kernel	Task
Previous modality	Every trial after a visual trial	Event kernel	Task
Previous success	Every trial after a successful trial	Event kernel	Task
Success	All successful trials	Event kernel	Task
Water given	All frames after a water reward was given	Event kernel	Task
Auditory stimulus (Left / Right)	All frames after a left- or rightward auditory stimulus	Event kernel	Task

Table 2: Number of mice included for each experiment

	**Optogenetics**	**Widefield**	**Two-photon**
Emx	9	4	-
CStr	9	4	-
Fezf2	8	5	-
PlexinD1	8	4	-
Camk2α-tTA;G6s2	-	-	3

Table 3: Resources

Reagent/resource	Source	Identifier
**Antibodies**		
Goat polyclonal anti-GFP	Abcam	ab6673
Donkey Anti-Goat Alexa Fluor 488	Abcam	ab150129
**Viral strains**		
CAV-2-Cre	Plateforme de Vectorologie de Montpellier	N/A
AAV1-hSyn1-SIO-stGtACR2-FusionRed	Penn Vector Core	105677-AAV1
AAVrg-CAG-tdTomato	Penn Vector Core	59462-AAVrg
AAV-DJ-hSYN-DIO-hCAR{off}<br>(Titer: 5.7e12 vg/ml)	Laboratory of Adam Kepecs	
**Experimental Models**		
Mouse: Emx1-IRES-Cre: Emx1$^{tm1(cre)Krj}$	The Jackson Laboratory	JAX#005628
Mouse: ROSA:LNL:tTA: Gt(ROSA)26Sor$^{tm1(tTA)Roos}$	The Jackson Laboratory	JAX#008600
Mouse: Camk2α-tTA: Tg(Camk2a-tTA)1Mmay	The Jackson Laboratory	JAX#003010
Mouse: Ai93(TITL-GCaMP6f)-D (Ai93D): Igs7$^{tm93.1(tetO-GCaMP6f)Hze}$	The Jackson Laboratory	JAX#024103
Mouse: Ai162(TIT2L-GC6s-ICL-tTA2)-D (Ai162D): Igs7$^{tm162.1(tetO-GCaMP6s,CAG-tTA2)Hze}$	H. Zeng, Allen Institute for Brain Science	JAX#031562
Mouse: TRE-GCaMP6s (G6s2): Tg(tetO-GCaMP6s)2Niell	The Jackson Laboratory	JAX#024742
Mouse: H2B-eGFP: Tg(HIST1H2BB/EGFP)1Pa	The Jackson Laboratory	JAX#006069
Mouse: Fezf2-2A-CreER: Fezf2$^{tm1.1(cre/ERT2)Zjh}$	The Jackson Laboratory	JAX#036296
Mouse: PlexinD1-2A-CreER: Plxnd1$^{tm2.1(flpo)Zjh}$	The Jackson Laboratory	JAX#036295
**Software**		
MATLAB 2018B	Mathworks	
Python 3.6.10	Python Software Foundation	
**Other**		
Bpod State Machine r0.5	Sanworks	N/A
