# Supplementary material for: Pyramidal cell types drive functionally distinct cortical activity patterns during decision-making
Source: Nat Neurosci. 2023 Jan 23;26(3):495–505. doi: 10.1038/s41593-022-01245-9 (PMC9991922; doi:10.1038/s41593-022-01245-9)
Supplement: Supplementary file 2 — Reporting Summary [file 41593_2022_1245_MOESM2_ESM.pdf]

nature portfolio

Corresponding author(s): Anne K. Churchland

Last updated by author(s): Jan 10, 2022

Reporting Summary

Nature Portfolio wishes to improve the reproducibility of the work that we publish. This form provides structure for consistency and transparency in reporting. For further information on Nature Portfolio policies, see our Editorial Policies and the Editorial Policy Checklist.

Statistics

For all statistical analyses, confirm that the following items are present in the figure legend, table legend, main text, or Methods section.

n/a	Confirmed	
☐	☒	The exact sample size (*n*) for each experimental group/condition, given as a discrete number and unit of measurement
☐	☒	A statement on whether measurements were taken from distinct samples or whether the same sample was measured repeatedly
☐	☒	The statistical test(s) used AND whether they are one- or two-sided<br>*Only common tests should be described solely by name; describe more complex techniques in the Methods section.*
☐	☒	A description of all covariates tested
☐	☒	A description of any assumptions or corrections, such as tests of normality and adjustment for multiple comparisons
☐	☒	A full description of the statistical parameters including central tendency (e.g. means) or other basic estimates (e.g. regression coefficient) AND variation (e.g. standard deviation) or associated estimates of uncertainty (e.g. confidence intervals)
☐	☒	For null hypothesis testing, the test statistic (e.g. *F*, *t*, *r*) with confidence intervals, effect sizes, degrees of freedom and *P* value noted<br>*Give P values as exact values whenever suitable.*
☒	☐	For Bayesian analysis, information on the choice of priors and Markov chain Monte Carlo settings
☐	☒	For hierarchical and complex designs, identification of the appropriate level for tests and full reporting of outcomes
☒	☐	Estimates of effect sizes (e.g. Cohen's *d*, Pearson's *r*), indicating how they were calculated

Our web collection on statistics for biologists contains articles on many of the points above.

Software and code

Policy information about availability of computer code

Data collection	Behavioral data was collected using Bpod r0.5, a commercially available data acquisition system (https://www.sanworks.io/shop/products.php?productFamily=bpod). Imaging data was collected using custom Matlab (2015b) software (for widefield data) and MScan 2.3 (commercially available through Sutter Instruments and used to acquire 2-photon data).
Data analysis	Data were analyzed using custom Matlab (2018b) code. As in previous papers, we will make all code available for public use via GitHub (https://github.com/churchlandlab).

For manuscripts utilizing custom algorithms or software that are central to the research but not yet described in published literature, software must be made available to editors and reviewers. We strongly encourage code deposition in a community repository (e.g. GitHub). See the Nature Portfolio guidelines for submitting code & software for further information.

Data

Policy information about availability of data

All manuscripts must include a data availability statement. This statement should provide the following information, where applicable:

- Accession codes, unique identifiers, or web links for publicly available datasets
- A description of any restrictions on data availability
- For clinical datasets or third party data, please ensure that the statement adheres to our policy

Provide your data availability statement here.

Field-specific reporting

Please select the one below that is the best fit for your research. If you are not sure, read the appropriate sections before making your selection.

☒ Life sciences ☐ Behavioural & social sciences ☐ Ecological, evolutionary & environmental sciences

For a reference copy of the document with all sections, see nature.com/documents/nr-reporting-summary-flat.pdf

Life sciences study design

All studies must disclose on these points even when the disclosure is negative.

Sample size	Sample sizes were selected based on our extensive knowledge of mouse-to-mouse variability in behavior (quantified in (Odoemene et al, 2018). This led us to include 54 animals in total. The number of animals for each cell type is listed in the Methods section. Sample sizes were based on previous studies of widefield/2-photon imaging (Musall*, Kaufman* et al, 2019) and optogenetics (Odoemene et al, 2018).
Data exclusions	All animals tested were included in the study.
Replication	For both encoding and decoding analyses, we report only cross-validated data; that is, the results from "held out" trials that are not used to fit the model parameters. This demonstrates that our results are not due to over fitting of the model. We also replicated previous results (Musall 2019) that demonstrate movements dominate neural activity in this new dataset (see Figure 4B). We did not perform additional replication experiments.
Randomization	Animals were presented with stimuli of randomized difficulty. In optogenetics experiments, stimulation trials were randomly interleaved (20% of trials). Animals were randomly selected for participation in widefield imaging vs. optogenetics experiments. Controls are all done within each animal (e.g., stimulating in primary visual cortex and in parietal cortex in the same animal) so that comparisons were not usually made across groups. When we did make comparisons across groups (e.g., optogenetics for PT vs. IT neurons), animals were assigned to each group based on their genetic background (e.g., whether they were Fezf2-creER or PlexinD1-creER mice).
Blinding	Experimenters were not blinded to which cell type expressed calcium indicators in a given mouse. However, the data collection process is entirely computer controlled and automatic so that experimenter's knowledge of the animal's genetic background was not able to influence stimulus presentation, stimulus difficulty, or any other experimental parameters. During data analysis, we often used existing pipelines (e.g., encoding and decoding models in Figs 4-6). Experimenters' were not blinded to group membership, but also had no opportunity to intervene because the analysis is entirely automated and is run in the same way for all subjects. For other analyses, e.g., sNMF and LocaNMF (Fig 2), we re-purposed existing analysis tools designed for other experiments (see S. Saxena et al, 2020). These analyses were not blinded, but the analysis consists of decomposing matrices into spatial and temporal components and there is no opportunity for the user to influence the outcome.

Reporting for specific materials, systems and methods

We require information from authors about some types of materials, experimental systems and methods used in many studies. Here, indicate whether each material, system or method listed is relevant to your study. If you are not sure if a list item applies to your research, read the appropriate section before selecting a response.

Materials & experimental systems

n/a	Involved in the study	
☐	☒	Antibodies
☒	☐	Eukaryotic cell lines
☒	☐	Palaeontology and archaeology
☐	☒	Animals and other organisms
☒	☐	Human research participants
☒	☐	Clinical data
☒	☐	Dual use research of concern

Methods

n/a	Involved in the study	
☒	☐	ChIP-seq
☒	☐	Flow cytometry
☒	☐	MRI-based neuroimaging

Antibodies

Antibodies used	For histological visualization of GCaMP6s, we used primary goat polyclonal anti-GFP antibody (1/500 dilution, Abcam ab6673) and secondary donkey anti-goat Alexa Fluor 488 (1/500 dilution, Abcam ab150129)
Validation	*Describe the validation of each primary antibody for the species and application, noting any validation statements on the manufacturer's website, relevant citations, antibody profiles in online databases, or data provided in the manuscript.*

Animals and other organisms

Policy information about studies involving animals; ARRIVE guidelines recommended for reporting animal research

Laboratory animals	All mouse strains were acquired from the Jackson Laboratory, the Allen Brain Institute, or generated at Cold Spring Harbor Laboratory. Transgenic strains crossed to generate double- and triple-transgenic mice used for imaging: Emx-Cre (JAX 005628), LSL-tTA (JAX 008600), Ai93D (JAX 024103), Ai162 (JAX 031562), G6s2 (JAX 024742) and H2B-eGFP (JAX 006069). EMX mice, used for calcium imaging, were bred as Ai93D;Emx-Cre;LSL-tTA. To avoid potential aberrant cortical activity patterns, EMX mice were on a doxycycline-containing diet (DOX), preventing GCaMP6 expression until they were 6 weeks or older. To obtain PT- and IT-specific transgenic lines, we used two inducible knock-in mouse lines (Fezf2-2A-CreER and PlexinD1-2A-CreER) that were generated by inserting a 2A-CreER or 2A-Flp cassette in-frame before the STOP codon of the targeted gene. Both strains have been extensively characterized to reflect endogenous gene expression patterns that are closely linked to specific excitatory neuron types and induce robust and uniform expression throughout the cortex. Only male animals were used. We have since designed a subsequent study including sex as a biological variable. Mice/rats were housed as breeding pairs or were weaned and housed by sex in individually ventilated autoclaved caging (Thoren Caging Systems, Hazelton, PA). Animals were maintained on sanitized cages and irradiated bedding with 1/4 inch corn cob bedding (The Andersons, Maumee, OH) and were fed a closed-formula, natural-ingredient, γ-irradiated diet (PicoLab Mouse Diet 5053, Purina LabDiet, St. Louis MO) ad libitum. A complete cage change was performed every 7-10 days within a biological safety cabinet (model Nu602-400Class II Type Nuaire, Plymouth, MN). The room was maintained on a 12:12-h light:dark cycle with a relative humidity of 30 – 70%, and room temperature ranging from 69-78oF.
Wild animals	The study did not involve wild animals.
Field-collected samples	The study did not involve samples collected from the field.
Ethics oversight	The Cold Spring Harbor Laboratory Animal Care and Use Committee approved all animal procedures and experiments. All surgical and behavioral procedures conformed to the guidelines, established by the National Institutes of Health.

Note that full information on the approval of the study protocol must also be provided in the manuscript.
