# Supplementary material for: Pyramidal cell types drive functionally distinct cortical activity patterns during decision-making
Source: Nat Neurosci. 2023 Jan 23;26(3):495–505. doi: 10.1038/s41593-022-01245-9 (PMC9991922; doi:10.1038/s41593-022-01245-9)
Supplement: Supplementary file 3 — Editorial Assessment Report [file 41593_2022_1245_MOESM3_ESM.pdf]

Contents of this report

1. **Manuscript details**: overview of your manuscript and the editorial team.
2. **Review synthesis**: summary of the reviewer reports provided by the editors.
3. **Editorial recommendation**: personalized evaluation and recommendation from all 3 journals.
4. **Annotated reviewer comments**: the referee reports with comments from the editors.
5. **Open research evaluation**: advice for adhering to best reproducibility practices.

About the editorial process

Because you selected the **Nature Portfolio Guided Open Access** option, your manuscript was assessed for suitability in three of our titles publishing high-quality work in your field of research. More information about Guided Open Access can be found here.

Collaborative editorial assessment

Your editorial team discussed the manuscript to determine its suitability for the Nature Portfolio Guided OA pilot. Our assessment of your manuscript takes into account several factors, including whether the work meets the technical standard of the Nature Portfolio and whether the findings are of immediate significance to the readership of at least one of the participating journals in the Guided OA pilot.

Peer review

Experts were asked to evaluate the following aspects of your manuscript:

- **Novelty** in comparison to prior publications;
- **Likely audience** of researchers in terms of broad fields of study and size;
- **Potential impact** of the study on the immediate or wider research field;
- **Evidence** for the claims and whether additional experiments or analyses could feasibly strengthen the evidence;
- **Methodological detail** and whether the manuscript is reproducible as written;
- Appropriateness of the **literature review**.

Editorial evaluation of reviews

Your editorial team discussed the potential suitability of your manuscript for each of the participating journals. They then discussed the revisions necessary in order for the work to be published, keeping each journal's specific editorial criteria in mind.

Journals in the Nature portfolio will support authors wishing to transfer their reviews and (where reviewers agree) the reviewers' identities to journals outside of Springer Nature.
If you have any questions about review portability, please contact our editorial office at guidedoa@nature.com.

Manuscript details

Tracking number	Submission date	Decision date	Peer review type
GUIDEDOA-22-00383	Jan 12, 2022	Apr 6, 2022	Single-blind

**Manuscript title**<br>Pyramidal cell types drive functionally distinct cortical activity patterns during decision-making<br><br>**Preprint:** https://www.biorxiv.org/content/10.1101/2021.09.27.461599v2	**Author details**<br>Anne Churchland<br><br>**Affiliation:** University of California, Los Angeles

Editorial assessment team

**Primary editor**	**Sachin Ranade**<br>Home journal: ***Nature Neuroscience***<br>ORCID: **0000-0002-5150-5776**<br>Email: **sachin.ranade@us.nature.com**
**Other editors consulted**	**David Rowland**<br>**Home journal: Nature**<br>**ORCID:**<br><br>**Cody Walters**<br>**Home journal: Nature Communications**<br>**ORCID:**
**About your primary editor**	Sachin received his PhD from Stony Brook University for his work on the responses of serotonin neurons in the dorsal raphe nucleus of rats engaged in an olfactory perceptual decision task. During his postdoctoral research at Cold Spring Harbor Laboratory he developed optogenetic tagging techniques to identify neural activity from distinct neuronal

	subtypes in mice to investigate their behavioral correlates. Prior to joining Nature Neuroscience, he was an editor at Nature Communications. His research interests include neural mechanisms of behavioral function and dysfunction at the circuit, systems, and computational level. Sachin is based in the New York office.

Editorial assessment and review synthesis

**Editor's summary and assessment**	The manuscript uses wide-field, 2-P calcium imaging and optogenetics to interrogate the dorsal cortex-wide activity dynamics of two distinct pyramidal neuron subtypes (PT, IT) during an auditory perceptual decision making task in mice.<br><br>Previous work has shown that IT and PT neuron subtypes have distinct functional roles. The advance here is in exploring the dynamics of the two pyramidal subtype defined subnetworks across the entire dorsal cortex.
**Editorial synthesis of reviewer reports**	The reviewer panel had 3 reviewers with overlapping expertise in perceptual decision making in mice, calcium imaging techniques and analysis of population dynamics. Reviewer 1 had a major concentration in analytical methods.<br><br>All reviewers' comments suggest that the manuscript has the potential to be impactful. The main concerns raised were related to potential limitations in the imaging approach, robustness of the analytical methods, and discussion of the present findings in light of previous results, including those by the authors. There was also a sense that the results are complex and need further clarification (mirroring our editorial outlook during the initial consideration of the manuscript)<br><br>As part of the Guided Open Access pilot, editors from Nature, Nature Neuroscience and Nature Communications have discussed the reviewer reports and the manuscript's suitability for our journals. After careful evaluation, our editorial recommendation is to revise the manuscript and submit back through the Guided Open Access submission portal for consideration at Nature Neuroscience or Nature Communications.

Editorial recommendation

Nature<br><br>Revision not invited	Following editorial assessment of the paper and reviewer reports it was felt that the conceptual advance is not sufficient for further consideration at Nature
Nature Neuroscience<br><br>Major revisions with extension of the work	We would expect the major revision to address all the technical concerns regarding the experiments and the analyses. We strongly encourage them to add data to address the conceptual concerns of Reviewer 3 to reconcile their data with previous work including their own.
Nature Communications<br><br>Major revisions	We would expect the major revision to address all the technical concerns regarding the experiments and the analyses.

Next steps

**Editorial recommendation 1:**	Our top recommendation is to revise and resubmit your manuscript to *Nature Neuroscience.*
**Editorial recommendation 2:**	You may also choose to revise and resubmit your manuscript to *Nature Communications.* This option might be best if the requested experimental revisions are not possible/feasible at this time.
**Note**	As stated on the previous page *Nature* is not inviting a revision at this time. Please keep in mind that the journal will not be able to consider any appeals of their decision through Guided Open Access.

Revision

To follow our recommendation, please upload the revised manuscript files using **the link provided in the decision letter**. Should you need assistance with our manuscript tracking system, please contact Adam Lipkin, our Nature Portfolio Guided OA support specialist, at guidedOA@nature.com.

Revision checklist

- ☐ Cover letter, stating to which journal you are submitting
- ☐ Revised manuscript
- ☐ Point-by-point response to reviews
- ☐ Updated Reporting Summary and Editorial Policy Checklist
- ☐ Supplementary materials (if applicable)

Submission elsewhere

If you choose not to follow our recommendations, you can still take the reviewer reports with you.

Option 1: Transfer to another Nature Portfolio journal
Springer Nature provides authors with the ability to transfer a manuscript within the Nature Portfolio, without the author having to upload the manuscript data again. To use this service, **please follow the transfer link provided in the decision letter.** If no link was provided, please contact guidedOA@nature.com.

> *Note that any decision to opt in to In Review at the original journal is not sent to the receiving journal on transfer. You can opt in to In Review at receiving journals that support this service by choosing to modify your manuscript on transfer.*

Option 2: Portable Peer Review option for submission to a journal outside of Nature Portfolio
If you choose to submit your revised manuscript to a journal at another publisher, we can share the reviews with another journal outside of the Nature Portfolio if requested. You will need to request that the receiving journal office contacts us at guidedOA@nature.com. We have included editorial guidance below in the reviewer reports and open research evaluation to aid in revising the manuscript for publication elsewhere.

Annotated reviewer reports

The editors have included some additional comments on specific points raised by the reviewers below, to clarify requirements for publication in the recommended journal(s). However, please note that all points should be addressed in a revision, even if an editor has not specifically commented on them.

Reviewer #1 information	
**Expertise**	Calcium imaging, computational neuroscience
**Editor's comments**	

Reviewer #1 comments	
**Section**	**Annotated Reviewer Comments**
**Remarks to the Author: Overall significance**	In this manuscript the authors use wide-field calcium imaging and optogenetics and to assess differential contributions of PT and IT neuron dynamics during a decision making task in mice. The results are interesting and it is exciting that the authors attempt to link specific motor and task information to different pyramidal cell types. The attempt to separate different PyN contributions to a complex task is novel and will be interesting to the community in the wider field.
**Remarks to the Author: Impact**	In general, I do feel that the paper has the potential to influence thinking in the field, but that it is currently limited by the complexity of the results. It appears difficult to pinpoint the difference between PT and IT neurons, and there are many confounding factors such as movement which dominates all PyN types. Also, while this paper is clear and well written (which I very much appreciate), the interpretation in the discussion is overly technical and it is hard to see the big picture.
**Remarks to the Author: Strength of the claims**	I have a few concerns regarding the quantitative methods used to establish a difference between IT/PT, as many of the results rely on specialised dimensionality reduction and decoding techniques outlined in previous papers but which are not standard practice. My first concern is whether the components identified using dimensionality reduction methods are really representing differences in PT/IT PyNs rather than, for example, different non-unique solutions or noise. It would help to show that the clustering of spatial filters is consistent across animals (see detailed point below), for example.<br><br>Second, it looks from the results like pretty much all of the activity is explained by movement (4b, movement is on the dashed line for full model's explained variance), and task variables aren't necessary to explain all the variance. There's an order of magnitude difference in the delta R^2 between movement and task, and the task variable with the highest delta R^2 is choice which is correlated with movement (lick). This confuses interpretation of the rest of the manuscript which focuses on task variables. In the optogenetic experiments, for example, how do we know these

	interventions are disrupting decision-making rather than movement initiation?
**Remarks to the Author: Reproducibility**	The techniques should be more clearly described in the methods section (the list below will give some examples of where the methods are not sufficiently clear).<br><br>- It's difficult to understand the rationale for switching between sNMF and localNMF. Is there a figure like 2b for localNMF to show whether the spatial regions identified are overlapping between PyN types?<br>- Why do the spatial components look so smooth ? (esp in 2c where as I understand there is no extra constraint beyond non negativity of the spatial filters)<br>- Why aren't the temporal dynamics from sNMF or localNMF ever analysed ? Why are they not constrained to be non-negative as well via regular NMF ?<br>- A schematic of the regressors used for the linear encoding model would be helpful for the reader, as well as which are considered " movement" vs "task" variables<br>- Why is choice included as a task variable rather than a motor variable? Isn't choice always reported by the same movement (lick in appropriate direction) ?<br>- A 1-sentence description of the MLE method used to choose the ridge regression penalty would be useful. Is it cross-validated? Are results sensitive to this parameter?<br>- As I understand, NMF components are not ordered by variance (unlike PCA components). Could the authors explain in the manuscript how they identified the components necessary to explain X% of the variance ?<br>- In the CV procedure, was shuffling performed over blocks of time to account for slow temporal correlations due to the slow calcium indicator?<br>- How was the L1 penalty parameter chosen for the logistic regression decoder?<br>- Why are there no statistics for the optogenetic experiments ? (Figure 8f)

Reviewer #2 information

**Expertise**	Calcium imaging, optogenetics, perceptual decision making
**Editor's comments**	

Reviewer #2 comments

Section	Annotated Reviewer Comments
**Remarks to the Author: Overall**	Musall et al examine the dynamics of distinct pyramidal cell types as well as their contributions to perceptual decision-making. The study makes use of two

**significance**	transgenic lines developed by the Huang lab to label intratelencephalic (IT) neurons and pyramidal tract (PT) neurons, together with EMX1-Cre line and an interesting retrograde viral strategy that labels cortico-striatal (CStr) neurons. The authors use these approaches to express GCaMP6 in distinct pyramidal cell types and perform widefield calcium imaging across dorsal cortex. Different pyramidal cell types show distinct cortex-wide dynamics. Using encoding and decoding models, the study finds that stimulus information is distributed across sensory, parietal, and frontal cortical regions, while choice information is enriched in frontal regions. Optogenetic inactivation of pyramidal cell types show that PT neurons in parietal cortex play the largest role during stimulus perception while all cell types in frontal cortex contribute to choice.<br><br>There is a revolution in the field with the taxonomy of pyramidal cell types being defined, but their functions during behavior are not yet well understood. This is a timely paper that examines the functional roles of two pyramidal cell types in decision-making. Differing from previous studies that focus on single brain regions, this paper examines these cell types comprehensively across dorsal cortex. The study is technically impressive and combines a number of cutting-edge approaches to gain insights into the dynamic and function of distinct cell types. There are many interesting findings in the paper. The selective involvement of parietal cortex PT neurons and differential involvements of parietal and frontal regions are both novel. Overall, the study is well done, and it will be a valuable addition to the literature. I have a few comments which could be addressed through additional analyses.
**Remarks to the Author: Impact**	The topic is timely and there are many interesting findings in the paper. This will be a valuable addition to the literature. Nature Neuroscience is appropriate for the manuscript.
**Remarks to the Author: Strength of the claims**	Major<br><br>1) The authors clearly show that the pattern of cortex-wide dynamics differ between IT and PT neurons (Fig 2). This is interpreted as these cell types forming “parallel sub-networks” and existence of “PyN-specific subregions within cortical areas”. However, I wonder if differential patterns of Cre expression in these lines might also contribute to different spatial patterns. Specially, if the density of Cre expression is not uniform across regions (not necessarily “missing” in certain regions which the authors try to control) and the patterns differ across the two lines, this could result in different spatial components by the decomposition analysis. Could the authors quantify the density of labeled neurons in these lines, particularly for the regions where spatial patterns differ?<br><br>2) The interpretation of choice signal for IT neurons in frontal cortical regions as measured by widefield calcium signal should warrant more caution. Several

places in the text mention a lack of choice activity in IT neurons. For example, page 10 “We found equally prominent choice signals in frontal cortex of PT mice while very little modulation was seen in IT mice (Fig. 6b, Supp. Fig. 5b,c).”; “Surprisingly, we also found a mild suppression of M2 in IT mice.”; page 11, “… a reduction of choice weights in IT neurons.” A lack of choice signal could, at least in part, be due to a mixed IT population containing neurons preferring either contralateral or ipsilateral choice. If the two types of neurons are present in equal proportions, this could result in a loss of choice selectivity in the summed signal. However, individual IT neurons could still be quite choice selective. The later 2-photon imaging experiment confirms this, showing individual neurons preferring either contralateral or ipsilateral choice (Fig 7). Optogenetic experiment also suggests that IT neurons in frontal cortex play indispensable roles in choice behavior (Fig 8). Yet, the text seems to suggest that IT neurons lack choice selectivity. Another example is on Page 11-12, “Earlier work suggested a lack of choice selectivity in intracortical projection neurons [REF 20, 21].” Together, this framing of the results may give readers the impression that IT neurons lack choice signal altogether whereas that may not be case. The cited studies in fact show IT population contains mixed contra and ipsi preferring neurons, but these studies do not report a lack of choice selectivity in IT neurons.

3) Several parts of the results are confusing, but these may be due to my misunderstandings. These could be better clarified for readers to avoid potential confusions.

a. Fig 2g legends states, “UMAP shows clustering of LocaNMF components from similar regions. ” How do individual brain regions map onto the UMAP? Do different islands on the UMAP correspond to different brain regions? There are 12 brain regions (Fig 2e), but many more islands on the UMAP.

b. Fig 2g, is the zoomed in UMAP in the gray box a rerun of UMAP on the components inside the bounding box on the left? I assume this is the case since the two look different. Why is rerunning necessary?

c. Fig 2i, how are the PyN-type-specific and unspecific components defined? Are the unspecific components simply the components from EMX line?

d. Fig 4a shows that task variables explain 0.1-0.3 R^2, but in Fig 4c for delta R^2, eliminating task variables only reduce R^2 by up to 0.03, ~10 times smaller. Is this due to correlations of movement and task variables? This is potentially confusing for readers.

e. Fig 4d, IT shows stronger encoding of stimulus whereas PT shows stronger encoding of choice. Are these differences statistically significant? Also, the lack of choice encoding in IT is confusing given its strong roles in behavior as revealed by optogenetic inactivation experiment (see major comment 2

above).

f. Fig 5a. PT has strong stimulus kernel. But Fig 4d shows that there is a lack of stimulus response.

g. Fig 7j histogram shows a higher fraction of CStr neurons with ipsi preference in ALM. But in Fig 7k, ipsi preferring and contra preferring neurons are both ~30% in ALM for CStr neurons. Why the discrepancy?

4) The relationship of widefield calcium signal to underlying neuronal activity may differ between IT and PT. IT neurons have more extensive axons in cortex, which may contribute a greater proportion of neuropil signal compare to PT neurons. In previous studies where widefield calcium signal is compared to neuronal activity (e.g. Makino & Komiyama 2017, Peters, Harris, Carandini 2021), the activity seems to closely reflect local spike activity. But these studies do not examine different cell types. Can the authors confirm whether the widefield signal of IT neurons is reflecting local somatic activity? The authors here have a valuable dataset of both cell-type specific widefield imaging and cellular-resolution imaging from 2 regions. Even though these imaging are not conducted simultaneously, perhaps this data can be leveraged to quantitatively compare the selectivity profile of widefield signal and somatic activity.

Minor

1) There is a large amount of choice signal in the barrel cortex (e.g. Fig 4c and Fig S5). The results mention this but without much explanation (e.g. page 9-10). How should this signal be interpreted? Is the choice signal statistically significant?

2) In Fig 6, the decoding weights span a large portion of dorsal cortex, covering somatosensory cortex and M1, although the frontal cortical regions clearly have stronger contributions in EMX and PT lines. To help interpret this distributed weights, can the authors provide some assessment of whether the weights are statistically significant?

3) The study presents an interesting approach to induce cortex-wide CStr expression using virus injections in the striatum in a hybrid GCaMP reporter line. Could the authors provide more coronal sections across the brain to show the full extent of the expression. This will be valuable data for others who want to adapt this approach.

4) The description of transgenic lines used in different experiments is somewhat confusing and can be better clarified. For example, "Camk2α-tTA-G6s2" referred to on page 13 is actually Camk2α-tTA crossed to G6s2. Also, is the Ai162;G6s2 hybrid reporter line crossed to the LSL-tTA line?

	5) What is AAV-DJ-hCAR used for in the viral approach to express Cre-dependent stGtACR2?<br><br>6) Fig 2g legend, “Black dots show UMAP location of example components in (e).” Is this referring to the components I, II, III in (i) instead of (e)?
**Remarks to the Author: Reproducibility**	Overall, statistical analyses are appropriate. A few additional comments on specific analysis are included above.

**Reviewer #3 information**	
**Expertise**	Calcium imaging, auditory perceptual decision making
**Editor’s comments**	

**Reviewer #3 comments**	
**Section**	**Annotated Reviewer Comments**
**Remarks to the Author: Overall significance**	This is a nice study that uses widefield and 2-photon calcium imaging and optogenetics to characterize the contributions of different pyramidal neuron types (IT and PT) throughout the dorsal cortex to auditory perceptual decision-making. The results are complex, as one might expect, given that they are based on large-scale datasets, and the authors have applied approaches and analyses of the data that are state-of-the-art and likely to serve as an example for other groups that are recording neural dynamics at cortex-wide scale. They find that PT and IT neurons exhibit different task-related responses, even within the same area, and their responses are of different dimensionality. They used a viral strategy to specifically target cortico-striatal projection neurons, and found that the dimensionality of the dynamics was intermediate between IT and PT cells, and their response profiles were distinct from both groups (indicating that the IT responses are not purely reflecting cortico-striatal responses). Cell-type specific optogenetic inactivations found that both frontal and parietal cortex were important for task performance. Intriguingly, parietal PT cells appeared to be critical for performance, both PT and IT cells in frontal cortex were important during the stimulus and delay periods, and frontal PT cells were unique in that they were important during the response period. The perturbation experiments support the conclusions based on analysis of neural dynamics that projection cell types appear to make unique contributions to

decision-making.

Overall, the data are of high quality, the analysis and presentation of the results are compelling and clear, and the work will be of interest to a reasonably broad audience. The fact that they corroborate the widefield results with 2-photon imaging, for one of the cell classes, is a major strength of the manuscript, and mitigated some of my concerns that the results might conflate somatic, dendritic, and axonal activity. My main reservation about the work is that there appear to be gaps in how it has been contextualized with respect to the broader literature. I also have some comments about interpretation.

(1) These very authors previously showed that inactivation of parietal cortex did not impair performance on an auditory perceptual decision-making task (Raposo et al., 2014; also see Erlich et al., 2015). However, here, they report effects of parietal inactivations on an auditory task. How are we to reconcile these discrepant results? Do the authors suspect it is purely due to species differences between rats and mice?

(2) Znamenskiy & Zador previously showed that cortico-striatal neurons in A1 are causally involved in a similar auditory decision-making task (although again, that study was performed in rats). Here, however, the authors do not find a role for cortico-striatal neurons in parietal cortex, and instead find that PT neurons in parietal cortex seem to be critical. Did the authors investigate the role of cortico-striatal neurons in A1, to determine if their results from parietal cortex generalize? Can they speculate about why the A1->striatum circuit is insufficient to support this behavior, and requires parietal cortex? Perhaps the key difference is that the Znamenskiy paper used tone frequencies, which are represented tonotopically in A1 and striatum and thus could be supported by cortical-striatal plasticity in accordance with the task rule, whereas the current study used click sequences, which would presumably not be represented by different populations of neurons in A1/striatum… In any case, if the authors have data that speaks to this issue, or can connect the dots between these studies in the discussion, that would improve the manuscript.

(3) The authors inactivate IT cells in parietal cortex and find that there is a negligible effect on behavior, but they previously showed that these cells are inhibited during the stimulus presentation and delay periods. Therefore, the lack of effect on behavior could just reflect a floor effect, where they are unable to inhibit the IT cells beyond their low activity levels during these epochs. Perhaps if they had performed a different perturbation (e.g., exciting them), they would have observed an effect on behavior.

(4) I believe they also show a suppression of activity of cortico-striatal cells in frontal cortex during the stimulus and delay periods. Could a similar floor effect explain why inhibiting these cells produces a weaker effect on behavior than the PT and IT cells?

	Figure 1d: is this the average over multiple mice? Or individual mice? If the latter, are these the same mice shown in panel c?<br><br>Minor comments:<br><br>Typo: page 16 "Differences between PyNs are therefore not only present with specific areas" should be within<br><br>Typo: page 17 "Focusing on PT and IT neurons, also allowed us target two" also allowed us to target two… plus I don't think the comma should be there.<br><br>I was intrigued by the finding that the PT and IT cell responses are of different dimensionality. Does that reflect the fact that there are more distinct cell types lumped together as IT cells? Or does it reflect encoding of additional variables? This seems beyond the scope of the current paper, but I would have appreciated a deeper dive into this result.
**Remarks to the Author: Impact**	The work adds to a growing body of literature that is relating cortex-wide activity, at the single-cell and widefield levels, to perceptual decision-making tasks in mice. While I am not sure there is a sufficient conceptual advance to warrant publication in Nature, the work is cutting edge and provides sufficiently novel results for the broad but more specialist readership of Nature Neuroscience.
**Remarks to the Author: Strength of the claims**	
**Remarks to the Author: Reproducibility**	The quality of the data and presentation appear to be high. They also state that they will make data and code freely available, which is excellent.

Open research evaluation

General information

Guidelines for Transparency and Openness Promotion (TOP) in Journal Policies and Practices ("TOP Guidelines")

The recommendations and requests in the table below are aimed at bringing your manuscript in line with common community standards as exemplified by the TOP Guidelines. While every publisher and journal will implement these guidelines differently, the recommendations below are all consistent with the policies at Nature Portfolio. In most cases, these will align with TOP Guidelines Level 2.

FAIR Principles

The goal of the recommendations in the table below related to **data or code** availability is to promote the FAIR Guiding Principles for scientific data management and stewardship (*Scientific Data* **3**: 160018, 2016). The FAIR Principles are a set of guidelines for improving 4 important aspects of digital research objects: **F**indability, **A**ccessibility, **I**nteroperability and **R**eusability.

ORCID

ORCID is a non-profit organization that provides researchers with a unique digital identifier. These identifiers can be used by editors, funding agencies, publishers, and institutions to reliably identify individuals in the same way that ISBNs and DOIs identify books and articles. Thus the risk of confusing your identity with another researcher with the same name is eliminated. **The ORCID website** provides researchers with a page where your comprehensive research activity can be stored.

Springer Nature collaborates with the ORCID organization to ensure that your research contributions (as authors and peer reviewers) are correctly attributed to you. Learn more at https://www.springernature.com/gp/researchers/orcid

Data availability
**Data Availability Statement**

**Other data requests**

All source data underlying the graphs and charts presented in the main figures must be made available as Supplementary Data (in Excel or text format) or via a generalist repository (eg, Figshare or Dryad). This is mandatory for publication in a Nature Portfolio journal, but is also best practice for publication in any venue.<br><br>The following figures require associated source data: Figure 7k
**Data publishing recommendations**
You may also be interested in publishing a Data Descriptor with Scientific Data to improve the usability of your data set. See the journal website for details: https://www.nature.com/sdata/<br><br>For more information about data publishing at Springer Nature, please see https://www.springernature.com/gp/authors/research-data/research-data-publishing
**Code availability and citation**
Thank you for making your custom code available via Github. Upon publication, Nature Portfolio journals consider it best practice to release custom computer code in a way that allows readers to repeat the published results. Code should be deposited in a DOI-minting repository such as Zenodo, Gigantum or Code Ocean and cited in the reference list following the guidelines described in our policy pages (see link below). Authors are encouraged to manage subsequent code versions and to use a license approved by the open source initiative.<br><br>See here for more information about our code availability policies: https://www.nature.com/nature-portfolio/editorial-policies/reporting-standards#availability-of-computer-code
**Ethics**

We believe that authors, peer reviewers and editors should be required to disclose any competing interests that might influence their decisions and conclusions around a particular piece of content. In the interests of transparency and to help readers form their own judgements of potential bias, Nature Portfolio journals require authors to declare any competing financial and/or non-financial interests in relation to the work described.<br><br>Please provide a 'Competing interests' statement using one of the following standard sentences:<br><br>1. The authors declare the following competing interests: [specify competing interests]<br>2. The authors declare no competing interests.<br><br>See the Nature Portfolio competing interests policy for further information:<br>https://www.nature.com/nature-research/editorial-policies/competing-interests<br>The Springer Nature policy can be found here:<br>https://www.springernature.com/gp/policies/editorial-policies
We believe that Springer Nature has a responsibility to support the relevant guidelines (based on research community or geographical region) that specify best practice in research and thus require all experimental results on animal and human participants to conform to the authors' local regulations and ethical standards, and we also encourage adherence to international standards.<br><br>Because your study uses live vertebrates, a statement affirming that you have complied with all relevant ethical regulations for animal testing and research is necessary. A statement explicitly confirming if the study received ethical approval, including the name of the board and institution that approved the study protocol is also required. The species, strain, sex and age of animals should be included.<br><br>Further details on our policies can be found at<br>https://www.nature.com/commsbio/editorial-policies/ethics-and-biosecurity
**Reporting & reproducibility**
**Materials availability**
**Data presentation**

When choosing a color scheme please consider how it will display in black and white (if printed), and to users with color blindness. Please consider distinguishing data series using line patterns rather than colors, or using optimized color palettes such as those found at https://www.nature.com/articles/nmeth.1618 The use of colored axes and labels should be avoided. Please avoid the use of red/green color contrasts, as these may be difficult to interpret for colorblind readers.
Bar graphs should only be used to present counts or proportions. If you are using bar graphs that present means/averages, it is best practice to include individual data points and/or convert the graph to a boxplot or dot-plot. You may wish to refer to this blog post (https://ecrlife420999811.wordpress.com/2018/07/10/beyond-bar-graphs-free-tools-and-resources-for-creating-more-transparent-figures-for-small-datasets/) about representing data distribution in plots (particularly for small datasets).
Please ensure that all microscopy images and photographs include a scale bar and this scale bar is defined on the panels or in the figure legends.
